# Harmonisation of welfare indicators for macaques and marmosets used or bred for research

**DOI:** 10.12688/f1000research.109380.1

**Published:** 2022-03-03

**Authors:** Mark J. Prescott, Matthew C. Leach, Melissa A. Truelove

**Affiliations:** 1 National Centre for the Replacement, Refinement and Reduction of Animals in Research (NC3Rs), London, NW1 2BE, UK; 2 School of Natural and Environmental Sciences, Newcastle University, Newcastle upon Tyne, NE1 7RU, UK; 3 Yerkes National Primate Research Center, Emory University, Atlanta, Georgia, GA 30329, USA

**Keywords:** behaviour, Callithrix, Macaca, non-human primate, refinement, welfare assessment, well-being, 3Rs

## Abstract

**Background: **Accurate assessment of the welfare of non-human primates (NHPs) used and bred for scientific purposes is essential for effective implementation of obligations to optimise their well-being, for validation of refinement techniques and novel welfare indicators, and for ensuring the highest quality data is obtained from these animals. Despite the importance of welfare assessment in NHP research, there is little consensus on what should be measured. Greater harmonisation of welfare indicators between facilities would enable greater collaboration and data sharing to address welfare-related questions in the management and use of NHPs.

**Methods: **A Delphi consultation was used to survey attendees of the 2019 NC3Rs Primate Welfare Meeting (73 respondents) to build consensus on which welfare indicators for macaques and marmosets are reliable, valid, and practicable, and how these can be measured.

**Results: **Self-harm behaviour, social enrichment, cage dimensions, body weight, a health monitoring programme, appetite, staff training, and positive reinforcement training were considered valid, reliable, and practicable indicators for macaques (≥70% consensus) within a hypothetical scenario context involving 500 animals. Indicators ranked important for assessing marmoset welfare were body weight, NHP induced and environmentally induced injuries, cage furniture, huddled posture, mortality, blood in excreta, and physical enrichment. Participants working with macaques in infectious disease and breeding identified a greater range of indicators as valid and reliable than did those working in neuroscience and toxicology, where animal-based indicators were considered the most important. The findings for macaques were compared with a previous Delphi consultation, and the expert-defined consensus from the two surveys used to develop a prototype protocol for assessing macaque welfare in research settings.

**Conclusions: **Together the Delphi results and proto-protocol enable those working with research NHPs to more effectively assess the welfare of the animals in their care and to collaborate to advance refinement of NHP management and use.

Research highlights **Scientific benefit(s)**

• Harmonises welfare indicators for macaques, enabling inter-lab comparative studies and also greater data sharing to boost sample sizes for welfare-focused research.
• Ranks welfare indicators and narrows the field for further investigation of those considered most important by experts.

**3Rs benefit(s)**

• Identifies context appropriate welfare indicators, that are valid, reliable and practicable, allowing better assessment of welfare, minimisation of harm and evaluation of the impact of refinement techniques.
• Potentially benefits the welfare of an estimated 100,000 non-human primates (NHPs) used globally per year in biomedical research.

**Practical benefit(s)**

• Presents a practical and generalised welfare assessment protocol to support laboratory staff in assessing, monitoring and maximising the health and wellbeing of macaques.

**Current applications**

• Welfare assessment of macaques bred for and used in research, including in toxicology, neuroscience, infectious disease and other disciplines.

**Potential applications**

• Welfare assessment of marmosets and other NHP species.
• Benchmarking of welfare standards/quality of life between facilities.

## Introduction

Globally, an estimated 100,000 non-human primates (NHPs) are used annually in biomedical research and testing, with a far larger number housed in breeding facilities ( Carlsson *et al*., 2004; Lankau *et al*., 2014; Zhang *et al*., 2014; Vermeire *et al*., 2017; Grimm, 2018). Accurate assessment of the welfare of these animals is essential for fulfilling ethical and legal obligations to minimise any harm caused by scientific or veterinary procedures, and for the effective implementation of refinement techniques such as analgesia and humane endpoints ( Rennie & Buchanan-Smith, 2006; Jennings & Prescott, 2009; Hawkins *et al*., 2011; Descovich *et al*., 2019). It is also important for evaluating enhancements to animal management aimed at promoting positive welfare states and good psychological well-being, such as environmental enrichment and training for cooperation with husbandry ( Chamove, 1989; Segal, 1989; Bassett *et al*., 2003; Lutz & Novak, 2005; Buchanan-Smith *et al*., 2005; Buchanan-Smith, 2010a; Coleman & Maier, 2010; Coleman & Novak, 2017). In some countries, there is a requirement for *in vivo* researchers to report to regulators the ‘actual severity’ experienced by the animals used in their experiments ( European Union, 2010; Home Office, 2014; USDA APHIS, 2018), which is predicated on the ability to recognise and accurately measure pain and distress. Welfare assessment is also a component of the scientific method, because physiological and psychological responses to suffering can significantly affect data quality ( Poole, 1997; Institute for Laboratory Animal Research, 2008). Minimising avoidable suffering is therefore necessary to ensure the validity of the scientific research performed ( Novak & Petto, 1991; Graham & Prescott, 2015; Hannibal *et al*., 2017; Prescott *et al.*, 2021).

Most NHP facilities have dedicated and highly trained animal care staff who go to great efforts to optimise the well-being of the NHPs in their care ( Coleman, 2011), and effective welfare assessment tools will enable them to better accomplish this. It is recognised that welfare assessment should encompass both physical health and psychological well-being ( National Research Council, 1998; Wolfensohn & Honess, 2005; Jennings & Prescott, 2009). However, working evaluations of laboratory NHP welfare are often based on measurements of various indicators presumed to be related to the extent of failure to cope, or difficulty in coping, with the environment ( Lutz *et al*., 1991; European Commission, 2002). Modern welfare assessments should also aim to evaluate positive as well as negative states of individuals ( Hawkins *et al*. 2011; Wolfensohn *et al*., 2018). Social play, allogrooming, food sharing, exploration, and relaxed gait have been suggested as behavioural indicators of positive NHP welfare in the laboratory, though relatively few have been validated ( University of Stirling, 2011; Blois-Heulin *et al*., 2015; NC3Rs, 2015; Ahloy-Dallaire *et al.*, 2018; Miller *et al*., 2020).

Most facilities that house or breed NHPs for research (i.e. laboratories, breeding centres, etc.) utilise a combination of animal-based indices, as this gives the best estimate of an individual NHP’s welfare state ( Novak & Suomi, 1988; National Research Council, 1998; Jennings & Prescott, 2009). These include physical or somatic observations (e.g. susceptibility to disease; growth rate; coat and body condition), physiological measurements (e.g. heart rate; body temperature; plasma cortisol), and structured behavioural assessments (e.g. behavioural repertoire; activity budgets; presence of quantitative or qualitative behavioural abnormalities) ( Poole, 1988; European Commission, 2002; Wolfensohn & Honess, 2005; Gottlieb & Pomerantz, 2021; Novak & Meyer, 2021). Some animal-based indices used in practice, such as stereotyped behaviour (e.g. pacing), have been criticised for their lack of validity or validation ( Poirier & Bateson, 2017; Polanco *et al*., 2021) and specificity ( Descovich *et al*., 2019). Regardless, animal-based indices can be used to assess the outcome of providing resources for animal care, such as cage space and a varied diet.

**Figure 1.**
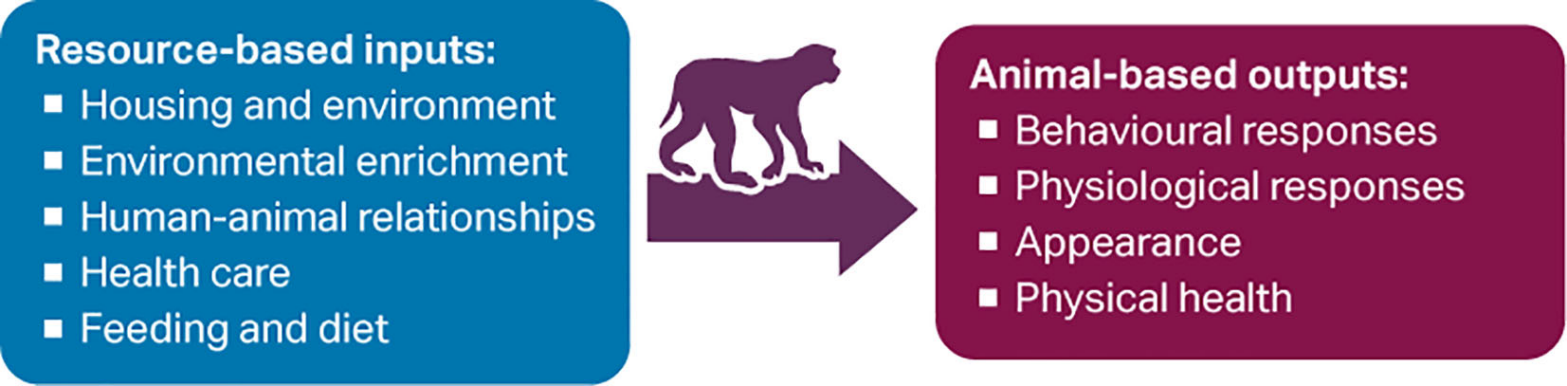
Some resource-based inputs and animal-based outputs that can be used to assess non-human primate (NHP) welfare.

A variety of resource-based indicators, which are variables measured not in the animals but in the environment, are also used to assess welfare (e.g. size and design of enclosures; provision of environmental enrichment; health monitoring programmes). These input-based, engineering criteria are attractive because they are objective, less time intensive, and easy to measure (e.g. during site inspection) ( Johnsen *et al.,* 2001; Mench 2003); however, they are often indirect measures of welfare and can be experienced differently by individuals (e.g. Izzo *et al.,* 2011; Velarde & Dalmau, 2012). Used alone, they do not effectively evaluate the welfare state of individual animals; but used alongside animal-based outcome indices, resource-based input indices can usefully contribute to welfare assessments, and are important for standardising within and between facilities, especially if founded on validated welfare needs ( Beaver & Bayne, 2014; Bennett *et al*., 2018) ( Figure 1).

Despite the importance of welfare assessment in NHP research, there are few established welfare assessment tools, and little is known about the level of consensus within the research community on whether the available indices are considered valid (i.e. genuinely measuring an aspect of an animal's welfare state), reliable (i.e. can be measured consistently across and between users), and practicable (i.e. can be measured with limited time, resources, and within facility constraints). Truelove *et al*. (2020) conducted a Delphi consultation to identify laboratory macaque welfare measures and their relative importance. A list of 115 potential indicators for use in welfare assessment of macaques (54 animal-based and 61 environment-based items) was provided to a panel of macaque experts, predominantly from North America. Experts indicated which indicators were valid, reliable, and practicable to measure using the provided on-site scenario ( Table 1a) and a composite percentage agreement score was assigned to each indicator, allowing subsequent ranking. Among the 39 experts who completed the two rounds of the survey, resource/environment-based measures were considered better suited than animal-based ones for on-site welfare assessment, with the presence of self-harm behaviours and provision of social enrichment considered the most important indicators for assessing macaque welfare; a total of 56 indicators were selected as being valid, reliable, and practicable. The ten indicators with the highest composite respondent percentage agreement score following two rounds of ratings included only one animal-based indicator (self-harm behaviour). These 56 indicators were presented as part of the current study, in part to gauge validity of the measures found in Truelove *et al*. (2020), as well as to uncover any indices that a different group of experts might accept or reject as useful in assessing macaque welfare.

**Table 1a.** Hypothetical welfare audit scenario.

You are participating in a welfare audit in an institution housing approximately 500 macaques. Individuals are housed indoors in 25 animal rooms which each hold 5 racks; each rack holds 4 cages and each cage houses 1 animal. Animals are either singly housed with access to one cage or are socially housed in pairs or groups with access to multiple adjacent cages (1 per animal) with a single rack; some individuals are participating in active research studies.

If there was a broader consensus on appropriate indicators of suffering and well-being in NHPs used for research, and widely applicable welfare assessment tools, then this would help researchers, veterinarians, and other animal care staff better fulfil their obligations to optimise the welfare of the animals in their care. Importantly, it could also facilitate greater collaboration and data sharing between research facilities to address welfare-related research questions, such as the impact of common procedures and putative refinements. Not only would this boost sample sizes for welfare-focused studies, especially those which must piggy-back onto ongoing scientific procedures conducted primarily for another research purpose, but it would also enable inter-laboratory comparative studies to identify how variation in management practices influence animal welfare ( Bliss-Moreau *et al*., 2021); doing so across an international audience might also identify practices diverging due to differences in culture and research specific to a region (e.g. McMillan *et al*., 2017; Baker & Prescott, unpublished work). In 2017, the United Kingdom’s national 3Rs centre (NC3Rs) led an international data crowdsourcing project to establish the prevalence and potential triggers for aggression-related injury in group-housed male laboratory mice ( Lidster *et al*., 2019) – a significant problem affecting the murine research community. In total, 143 animal technicians from 44 facilities collected aggression and husbandry data on over 137,000 mice using a common data collection framework. By comparing the prevalence of aggression and husbandry variables between facilities, the key factors that influence levels of aggression in male mice were identified, leading to recommendations for practical changes to husbandry to minimise aggressive behaviour and improve mouse welfare. This work illustrates the potential for welfare improvements when tapping into the expertise of a large group, regardless of the approach taken (e.g. crowdsourcing, Delphi).

To achieve broad consensus for NHP welfare indicators, and to develop a practical protocol for assessing macaque welfare, advantage was taken of the assembly of a group of NHP experts at the 2019 NC3Rs Primate Welfare Meeting. This international event supports laboratory and breeding centre staff working directly with NHPs to develop, share, and implement evidence-based refinements in NHP use and care. At the 2019 meeting, a hybridised Delphi consultation was undertaken to help harmonise NHP welfare assessment by gaining agreement amongst the experts on a list of welfare indices for macaque and marmosets that are valid, reliable, and practicable. Additionally, participants were surveyed about the methods used to measure each of these indices.

A classical Delphi consultation is an iterative, multi-stage survey technique that involves controlled feedback to a panel of anonymous subject experts; the consultation results in statistical group consensus on a selected topic as indicated by response stability between rounds ( Van Zolingen & Klaassen, 2003). This is in contrast to the group Delphi/expert workshop approach, in which a panel of experts work together, rather than independently, on a topic to arrive at consensus ( Webler *et al*., 1991) – all other elements are identical. We integrated both approaches for this study, using a classical Delphi in one round and a group Delphi in another round. Achieving consensus between experts increases the validity of the welfare assessment protocol and ensures that it incorporates a wide range of expert opinions, so that it is not perceived as an imposition from a single group of people ( Boulkedid *et al*., 2011).

## Methods

Online survey software from Qualtrics was used to survey the delegates of the NC3Rs Primate Welfare Meeting (8 November 2019, London) about their views on welfare indices for macaques and marmosets, as part of a hybridised Delphi consultation process. The inclusion criteria were being a delegate of the meeting and being directly involved in the care, use or breeding of NHPs for research, which all participants met. The survey was constructed and administered by the authors. Participation was voluntary and responses were submitted using personal mobile devices. The link to the survey was emailed on the day of the meeting and also displayed at the event. Participants completed a consent statement online at the start of the survey. Additionally, if any participant wished to withdraw consent at any time, they were asked to contact the NC3Rs team who would then remove the data they had supplied. All delegates provided consent and no delegates subsequently retracted consent. Quasi-anonymity was maintained: responses remained unknown to other participants but were known to the authors and response data were coded by username after receipt so that individuals’ responses could not be readily linked. Data collection procedures were approved by the Ethics Committee of the Faculty of Science, Agriculture and Engineering at Newcastle University. All data were managed according to a data management plan for NC3Rs office-led data sharing projects.

Participants were researchers, veterinarians, and animal technologists working directly with NHPs in nine countries (United Kingdom [UK], France, Germany, Hungary, Italy, Netherlands, Sweden, Switzerland, and the United States of America [USA]), with three-quarters based within the UK. Respondents were asked to identify their species of focus (macaque or marmoset) and area of specialty (neuroscience, infectious disease, toxicology, breeding, or other). In this way, we were able to actively control for species as a potential source of bias in the study. The survey method (hybridized Delphi) also addressed two potential biases (dominance effect and Von Restorff effect) through the use of multiple rounds and anonymity ( Hallowell, 2009).

**Figure 2.**
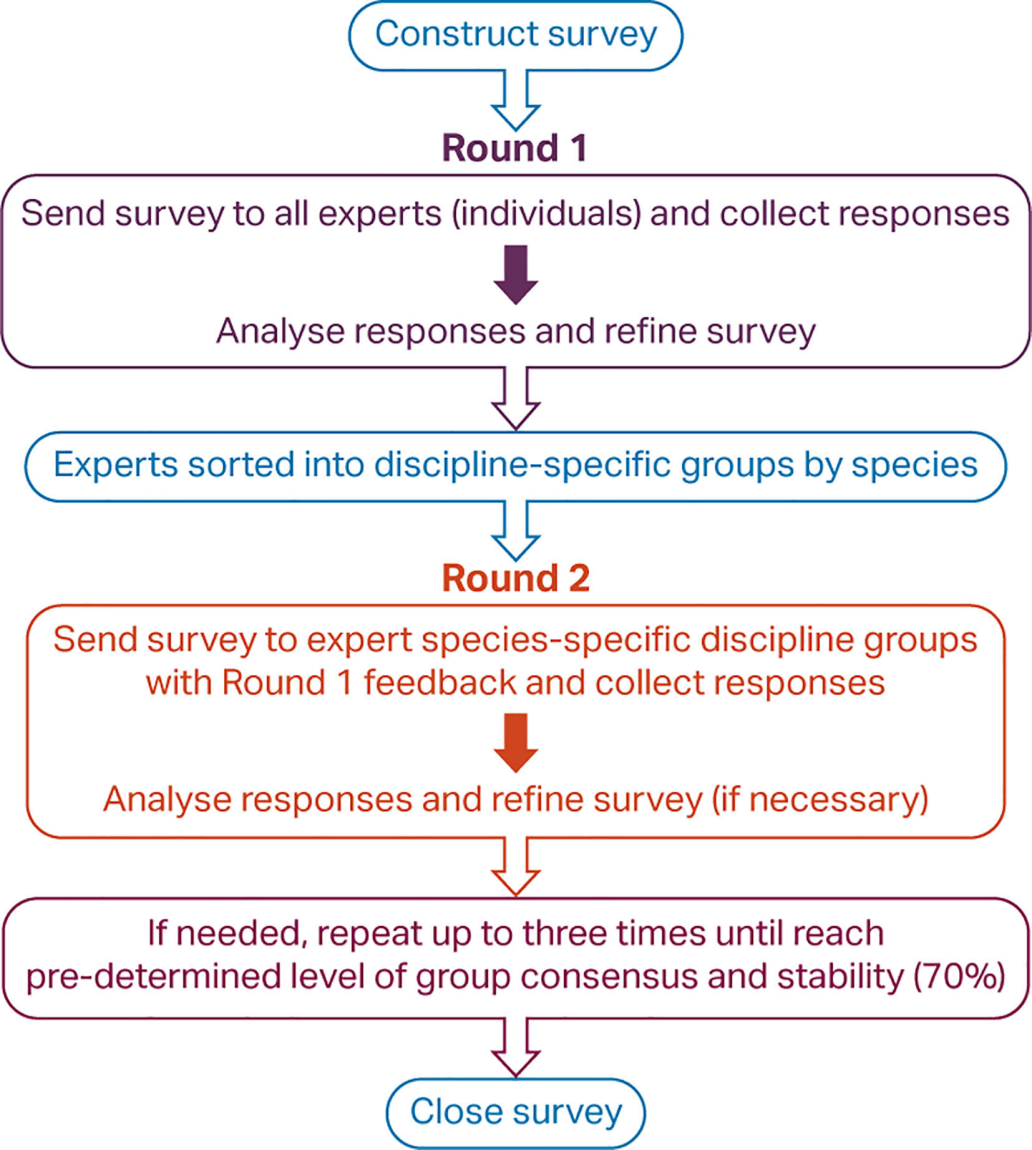
Steps in a hybridised Delphi process.

Multiple steps were required to complete the hybridised Delphi consultation process ( Figure 2). First, participants were presented with the scenario in Table 1a. They were then presented with the top ten welfare indices identified as valid, reliable, and practicable (≥70% consensus) for assessing macaque welfare in the Delphi consultation of Truelove *et al*., 2020 ( Table 1b) and asked:

**Table 1b.** Top ten indicators from Truelove *et al*. (2020).

Human euthanasia programme
Health monitoring programme
Food enrichment
Physical enrichment
Social enrichment
Self-harm behaviour
Ventilation
Behavioural management programme
Hear other NHPs
Cage furniture

Q1 “ *Which of the following indices do you think are the most valid and reliable for assessing NHP welfare? (select as many indices as you feel are appropriate)”*

Q2 “ *How practical are the indices you selected for assessing NHP welfare from the top ten?*” (with the options: “ *Very impractical; Impractical; Neither; Practical; Very practical*”)

Next, the participants were presented with a more extensive list of 56 welfare indices from the aforementioned Delphi consultation and asked:

Q3 “ *Of the 56 indices, which do you think are the most valid and reliable for assessing NHP welfare? (select as many indices as you feel are appropriate)*”

Q4 “ *How practical are the indices you selected for assessing NHP welfare from the 56 indices?*” (with the options: “ *Very impractical; Impractical; Neither; Practical; Very practical*”)

Finally, participants had the opportunity to suggest additional indices that they considered to be valid, reliable, and practicable for assessing welfare (Q5; free text responses; 5% threshold).

In a second round of the consultation, the participants were split into pre-assigned groups according to the scientific disciplines they worked in and whether their work involved marmosets or macaques. Working as a group and bearing in mind the same scenario ( Table 1a), participants were provided feedback from the first round as to which welfare indicators were considered valid and reliable, and which were considered practicable. They were asked to discuss and then define how they would measure each of the indices identified as being valid, reliable, and practicable in round 1 (i.e. at or exceeding 70% consensus, as per Truelove *et al*., 2020 and Leach *et al*., 2008). Specifically, they were asked to consider the following and then respond as a group:

Q6 “ *Are you recording this measure at an individual, group/cage, room or unit level?*” (with the option: “ *Other [please specify]*”)

Q7 “ *How would you record this measure? i.e. what method and equipment (if any) would you use?*” (free text responses)

Q8 “ *How long would you spend recording this measure? i.e. would you measure this intermittently, and how frequently or constantly and over what period of time etc.?*” (free text responses)

Q9 “ *What proportion of animals/groups/rooms/units would you assess in order to get a meaningful assessment?*” (selected choice in 10% intervals from <10% to 100%; with the option: “ *Other [please specify]*”)

The top ranked welfare indicators for macaques identified during the two Delphi consultations and the information obtained regarding their measurement was then used, along with the expertise of the authors, to construct a prototype protocol for assessing macaque welfare in research settings.

### Statistical analysis

Many Delphi studies have used percentage measures as their primary indication of consensus, despite disagreement as to whether this is adequate ( Hsu & Sanford, 2007). We set an *a priori* agreement level of 70% or greater for consensus, as has been done in other animal welfare and healthcare studies (e.g. Leach *et al*., 2008; Keeney *et al.,* 2011).

Descriptive statistics were used to summarize the participants’ responses per round. For all completed surveys, there were no missing data. For those surveys started but without any collected data (i.e., no answers provided but an identifier was issued), these were removed so as to not inflate the number of participants. Data were imported into Microsoft Excel for Microsoft 365 (2021) and summarised for analysis; participant identifiers were removed to maintain anonymity. Free text comments were analysed qualitatively and were grouped by similar idea by one coder (MAT).

To complete the Delphi process, group stability (i.e. consistency of response between rounds) must be demonstrated ( von der Gracht, 2012); this was achieved by Krippendorff’s alpha coefficient (α) test ( Hayes & Krippendorff, 2007 *).* For interpretation, a value of 0 indicates perfect disagreement whereas 1 indicates perfect agreement; a value of 0.667 or more permits (tentative) conclusions to be made ( Krippendorff, 2004).

## Generalised macaque welfare assessment protocol (GEN-MAC)

Our generalised macaque welfare assessment protocol aims to offer a practical and context appropriate tool for laboratory staff caring for macaques ( Table 2). It provides a quantitative set of criteria to support staff in monitoring and maximising macaque health and well-being, based on expert consensus. The tool encompasses all four domains of potential welfare compromise (i.e. nutritional, environmental, health, behavioural) identified by the Welfare Quality® project ( Blokhuis *et al*., 2013) and Mellor *et al*. (2009). Taken together, the chosen indicators should provide an assessment of an individual animal’s welfare, and hence, when repeatedly measured over time, provide an assessment of its quality of life ( Fraser, 2008). We acknowledge that good animal welfare is more than the mere absence of negative experiences and recognise that the tool incorporates few indicators of positive welfare state currently; however, validation of these is proving difficult (e.g. see Ahloy-Dallaire *et al*., 2018 for a discussion of the relationship between play and positive affective states). As new indices of positive state are validated, they can be incorporated into this tool.

**Table 2.** Generalised welfare assessment protocol for laboratory-housed macaques.

Assessor: ___________ Animal ID: ___________ Date: ___________			
Indicator	Score	Scoring criteria	References
***Animal-based – score for each animal***			
**1. Self-harm behaviour** *E.g. on inspection or recorded in daily logs. Where seen, more frequent and detailed follow-up observations can be made to assess incidence, severity, and impact.*	0	Self-harm behaviour not observed; individual not known to self-harm	Reinhardt & Rossell, 2001; Novak 2003, 2021; Polanco *et al*., 2021
	1	Self-harm behaviour observed, without physical injury; individual known to self-harm occasionally (e.g. self-biting, self-hitting, eye poking, head-banging, hair plucking/pulling)	
	2	Physical injuries present, consistent with self-harm behaviour (e.g. abrasions, lacerations, eye trauma)	
**2. NHP induced injuries** ***Use 2a and 2b.*** 2a. Injuries *E.g. on inspection or recorded in daily logs.*	0	No injuries present	Beisner *et al*., 2019; Crast *et al*., 2021
	1	Minor injuries present, consistent with fighting; may or may not require veterinary intervention/treatment (e.g. abrasion/blunt trauma, puncture wound, skin laceration)	
	2	Severe injuries present, consistent with fighting; veterinary intervention/treatment necessary (e.g. deep or multiple laceration/s, skin puncture + muscle involvement, inflamed or infected wound, bone exposure, degloving; signs of pain such as grimacing, hunched posture, guarding of limb)	
2b. Lameness *E.g. on inspection or recorded in daily logs.*	0	No signs of lameness or imbalance when moving; unimpaired/normal locomotion	Lewis & Colgin, 2005; Wren *et al.*, 2013
	1	Signs of lameness or imbalance when moving (e.g. limping or favouring limb, slow locomotion or uneven rhythm, unable to keep up with the group, guarding of limb); interfering with normal locomotion, may or may not require veterinary intervention/treatment	
	2	Persistent or long-term signs of lameness or imbalance when moving; inability to locomote normally; requires veterinary intervention/treatment	
**3. Appetite** ***Choose the most appropriate method for your context: 3a, 3b or 3c for eating, plus 3d or 3e for drinking.*** 3a. Hand feeding *For animals that are typically comfortable taking food from the hand.*	0	Takes preferred food	Keeling & Wolf, 1975; Wolfensohn & Honess, 2005; Smith *et al*., 2006; Prescott *et al*., 2010; Association of Primate Veterinarians, 2019
	1	Takes only small amounts of preferred food	
	2	Refuses to take preferred food; reluctant to come forward	
3b. Eating habits *E.g. on inspection at feeding time; or recorded in daily logs.*	0	Observed to eat normally; evidence of food consumption (e.g. crumbs/scraps)	
	1	Observed to not eat; no evidence of food consumption (e.g. no crumbs/scraps or food intact)	
	2	Repeatedly observed to not eat (i.e. for more than one day); protracted lack of evidence of food consumption (e.g. no crumbs/scraps or food intact)	
3c. Food consumption *E.g. in the last 24 hours; weigh food or count biscuits/residues); can be recorded at the group level.*	0	Normal amount of food consumed	
	1	Reduced food consumption (e.g. 25-75% relative to baseline; or 1 SD below the mean)	
	2	No (or very little) food consumed; reduced faecal output	
3d. Drinking habits *E.g. on inspection at feeding time; or recorded in daily logs.*	0	Observed to drink normally; evidence of water consumption (e.g. approach water source)	
	1	Observed to not drink; no evidence of water consumption (e.g. does not approach water source)	
	2	Repeatedly observed to not drink (i.e. for more than one day); protracted lack of evidence of water consumption (e.g. does not approach water source)	
3e. Fluid consumption *E.g. in the last 24 hours; weigh water bottle; can be recorded at the group level.*	0	Normal amount of fluid consumed
	1	Reduced fluid consumption (e.g. 25-75% relative to baseline; or 1 SD below the mean)
	2	No (or very little) fluid consumed; dry faeces
**4. Body weight *Use 4a or 4b (age dependent), plus 4c.*** *Note weight loss is expected for some research protocols.* 4a. Body weight change (for adult animals) *E.g. relative to previous day or week.*	0	No change in body weight	Wolfensohn & Honess 2005; Smith *et al*., 2006
	1	<10% change in body weight (or 1SD from expected for individual of that age) *(Weight loss over a certain period may not be a concern in overweight animals, so check body condition score, 4c)*	
	2	>10% change in body weight (or 2SD from expected for individual of that age)	
4b. Growth rate (for young, growing animals) *E.g. using colony-specific growth curves, or those in the published literature.*	0	Appears to be growing normally (e.g. body weight within normal range for age and sex, growth is following the relative centile)	Van Wagenen & Catchpole, 1956; Prescott *et al*., 2010
	1	Deviation from expected growth rate (e.g. body weight outside normal range for age and sex, deviation from the individuals’ normal growth trajectory, crossing centiles)	
	2	Ceasing to grow normally (e.g. body weight far outside normal range for age and sex)	
4c. Body condition score *Condition scoring may be performed non-invasively through observation, or during clinical examination by palpating the thoracic and lumbar vertebrae.*	0	Body condition score of 3 (normal/optimal)	Wolfensohn & Honess, 2005; Clingerman & Summers, 2012
	1	Body condition score of 2 (underweight/thin) or 4 (overweight/heavy)	
	2	Body condition score of 1 (severely underweight/emaciated) or 5 (obese/grossly obese)	
**Sub-total: _ /14**			
***Resource-based – score for the cage***			
**5. Social enrichment** ***Use 5a and 5b.*** 5a. Social condition *Regardless of whether an exemption from social housing is approved by the IACUC/AWERB.* *In very rare cases, individuals may thrive without a social partner.*	0	Continuously socially housed with one or more compatible conspecifics in the same cage/enclosure	Schapiro *et al.,* 1996; Lutz & Novak, 2005; Gilbert & Baker, 2011; Baker *et al*., 2012, 2014; DiVincenti & Wyatt, 2011; Hannibal *et al*., 2017; Cassidy *et al*., 2020
	1	Intermittent social housing (during part of the day/week)	
	2	Single housed (no physical contact but visual and olfactory contact provided); OR protected contact (separation of individuals via a barrier that permits social contact but not entry into each other’s cage/enclosure)	
	3	Social isolation (no sensory contact with conspecifics).	
5b. Social behaviour *E.g. on inspection or recorded in daily logs.*	0	Frequent prosocial/affiliative interactions observed between social partners; pair/group appears stable	
	1	Both affiliative and agonistic interactions observed between social partners; may be some signs of pair/group instability	
	2	Frequent agonistic interactions and few affiliative interactions observed between social partners; pair/group appears unstable; OR animal singly housed	
**6. Caging environment** ***Use 6a and 6b.*** 6a. Cage dimensions *Assessed against, e.g. ILAR Guide; ETS 123; Directive 2010/63/EU.*	0	Provided with more than the regulatory/accreditation-related minimum space allowance (e.g. via large indoor enclosure, outdoor enclosure, access to additional exercise enclosure/play pen)	Reinhardt *et al.*, 1996; Buchanan-Smith *et al*., 2004; Griffis *et al*., 2013
	1	Provided with the regulatory/accreditation-related minimum space allowance	
	2	Provided with less than the regulatory/accreditation-related minimum space allowance (e.g. metabolism cage)	
6b. Vertical space	0	Housed in cage floor to ceiling high, with adequate high perching, verandas, etc. to allow all occupants to move to heights about human eye level	Reinhardt, 1992; Nakamichi & Asanuma, 1998; Reinhardt, 2003; Clarence *et al*., 2006; Maclean *et al*., 2009; Gottlieb *et al.,* 2014; Lutz & Brown, 2018
	1	Housed in cage floor to ceiling high, with limited high perches, verandas, etc., meaning not all animals have access	
	2	Housed in double-tiered (1-over-1) caging that lacks perching at or above human eye level	
**7. Physical enrichment (including cage furniture)** ***Use 7a and 7b.*** 7a. Provision of physical enrichment	0	Complex environment, with ample physical enrichment provided including structural enhancements (e.g. swings, ladders, shelves, tyres, hammocks, perches) that allow for species-typical locomotion (climbing, leaping, running, etc.), visual barriers, and manipulanda (e.g. toys, mirrors, wood blocks); structural complexity allows for as much of the housing to be used as possible	Bryant *et al*., 1988; Turner & Grantham, 2002; Honess & Marin, 2006; Waitt *et al.,* 2008, 2010; Griffis *et al*., 2013; Descovich *et al.,* 2019
	1	Limited physical enrichment provided (e.g. perches with toys)	
	2	No physical enrichment provided	
7b. Use of physical enrichment *E.g. on inspection or recorded in daily logs.* *Note use of physical enrichment can vary with age.*	0	Observed to frequently interact with physical enrichment in species-typical, positive way	
	1	Observed to occasionally interact with physical enrichment in species-typical, positive way	
	2	No interaction (or abnormal interaction) with physical enrichment	
**8. Food enrichment**	0	Food presentation encourages daily and extended bouts of species-typical foraging behaviour (e.g. scatter feeding of fine forage mix into floor substrate; feeding fresh browse and edible plants from approved sources; utilising puzzle feeders, which require considerable time, manipulation, and fine motor skills for retrieval of food)	Chamove *et al*., 1982; Boccia 1989a,b; Byrne & Suomi, 1991; Reinhardt, 1994; Doane *et al*., 2013
	1	Food presentation encourages species-typical foraging behaviour, but such opportunities are not daily, nor extended (e.g. feeding whole fresh produce)	
	2	Food presentation does not allow species-typical foraging behaviour (e.g. cafeteria-style presentation in bowls)	
**Sub-total: _ /15**			
***Staff-based – score for the individual or facility, as appropriate***			
**9. Positive reinforcement training (PRT)** ***Use 9a and 9b.*** 9a. Individual training performance *E.g. on inspection or recorded in daily logs.*	0	Individual is trained, using positive reinforcement, to voluntarily cooperate with the scientific, veterinary, and husbandry procedures it is exposed to; it reliably cooperates, showing a high degree of compliance and few signs of distress	Prescott *et al*., 2005; Prescott & Buchanan-Smith, 2007; Perlman *et al*., 2012; Baker, 2016; McMillan *et al.,* 2017
	1	Individual is trained, using positive reinforcement, to voluntarily cooperate with the scientific, veterinary and husbandry procedures it is exposed to; however, it does not reliably cooperate and shows signs of distress	
	2	Individual is not trained to voluntary cooperate with procedures	
9b. PRT programme		Characteristics of a high-quality programme: a. A specialist in behaviour modification was involved in development of the training programme b. One person has overall responsibility for the training programme (training coordinator) to ensure a consistent approach c. Animals are trained by dedicated staff members, who possess a high degree of training knowledge and competence d. Training procedures are documented in SOPs/protocols e. Methods are predominantly based on PRT; where negative reinforcement is used, it is in combination with PRT and only where PRT alone has failed f. The training programme allows sufficient time for progressive habituation to conditions, techniques, and procedures before data collection begins g. Records of individual training performance are kept and regularly reviewed; training is tailored to individual differences in learning	
	0	Programme includes more than four of the above characteristics	
	1	Programme includes two to four of the above characteristics	
	2	Programme includes less than two of the above characteristics	
**10. Behavioural management programme**		Characteristics of a high-quality programme: a. Incorporates enrichment interventions that satisfy a range of needs in the social, locomotory, sensory, cognitive, and food-based domains b. Offers novelty (objects, space, activities) to stimulate and provide new challenges c. Provides animals with a degree of choice and control in their environment d. Incorporates regular monitoring for behavioural signs of distress, including prolonged withdrawal or hunched posture, prolonged expression of stereotypies, or excessive fearful behaviours e. Overseen by a dedicated individual with behavioural science or veterinary expertise f. Behavioural management strategies/procedures are documented in SOPs/protocols g. Behavioural management forms part of research protocol review	Bloomsmith, 2017; Bloomsmith *et al*., 2018; Schapiro, 2021
	0	Programme includes more than four of the above characteristics	
	1	Programme includes two to four of the above characteristics	
	2	Programme includes less than two of the above characteristics	
**11. Humane euthanasia programme** ***Use 11a and 11b.*** 11a. Euthanasia programme	0	Personnel responsible for carrying out euthanasia are knowledgeable and competent to perform the procedure in a compassionate, professional, and appropriate manner that avoids distress to the animals; AVMA-approved methods are used	Canadian Council on Animal Care, 2019; Lambeth *et al*., 2013; American Veterinary Medical Association, 2020
	2	Personnel responsible for carrying out euthanasia are not suitably trained and competent; euthanasia methods are not AVMA-approved	
11b. Humane endpoints		Characteristics of a high-quality programme: a. Accurate and clearly defined humane endpoints are established prior to study initiation b. Animals are monitored at an appropriate time and frequency to enable the earliest possible euthanasia decision and avoid moribundity/spontaneous death c. Humane endpoints are regularly reviewed and refined over time, as new data becomes available from studies using the model d. All personnel performing endpoint criteria assessments are able to recognise the signs of ill health e. Structured welfare assessment/humane endpoint score sheets are available and included in SOPs f. Where applicable, technologies such as telemetry, actimetry, imaging, and CCTV are used to inform endpoints decisions	Association of Primate Veterinarians, 2020; Prescott *et al*., 2021
	0	Programme includes more than four of the above characteristics	
	1	Programme includes two to four of the above characteristics	
	2	Programme includes less than two of the above characteristics	
**12. Health monitoring programme**	0	Comprehensive health monitoring programme is in place, (e.g. as specified by FELASA), to track colony health and prevent disease outbreaks	Balansard *et al*., 2019
	2	No comprehensive health monitoring programme	
**13. Staff training** ***Use 13a and 13b.*** 13a. Staff training progamme	0	All relevant staff (research, veterinary, and animal care) undergo a structured training programme before working with macaques; individual training records are kept and regularly reviewed (e.g. annually)	Wolfensohn & Honess, 2008; Jennings & Prescott, 2009
	1	A structure programme is in place, but records are not kept or regularly reviewed	
	2	No structured programme in place	
13b. Continuing professional development	0	All relevant staff (research, veterinary, and animal care) have the opportunity to attend internal and/or external presentations, conferences, and/or workshops on macaque welfare, care, and behaviour (e.g. NC3Rs Primate Welfare Meeting)	
	2	Staff do not have access to continuing professional development opportunities	
**Sub-total: _ /16**			
**Grand total: _ /45**			
**Judgement:** 0-15: Normal; assume good welfare state. 16-30: Welfare compromised; improvements required; monitor carefully. 31-45: Welfare severely compromised; suffering is likely; immediate action required; provide appropriate examination, treatment, and relief (e.g., analgesia, environmental adjustments).			

This tool is not intended to replace welfare assessment protocols tailored to specific scientific disciplines, projects, procedures, and adverse effects. Rather it presents an appropriate number of valid, reliable, and practicable indicators for a generalised assessment of “wellness” that can inform and augment existing specific tools. This generalised tool is particularly suited for high level assessments of the outcome/quality of institutional behavioural management programmes and comparisons between laboratories. Where appropriate, facilities working in specific disciplines may wish to supplement this core set of indicators with additional ones listed in Figure 4 or the literature (e.g. Smith *et al*., 2006; Wolfensohn & Honess, 2005; Honess & Wolfensohn, 2010; Kirchner & Bakker, 2015; Descovich *et al*., 2019 for neurophysiology). Where physiological measurements are required, the least invasive and most refined method that will provide the necessary data should be used (e.g. Davenport *et al*., 2006; Rennie & Buchanan-Smith, 2006; Smiley Evans *et al*., 2015). Awareness of the context for the assessment is important; for example, food intake can be reduced following administration of anaesthetic and analgesic medication, as well as due to pain or illness.

Like other welfare assessment tools, this one combines animal-based measures of welfare with indirect resource- and staff-based ones, which are more amenable for assessing the welfare of large numbers or groups of animals when under time constraints. There is evidence that the resource- and staff-based measures included are closely associated with outcomes indicative of good animal welfare in macaques, even if they do not guarantee that any one animal is experiencing a good quality of life ( Jennings & Prescott, 2009; Schapiro *et al*., 2014). For example, it can be time consuming to measure affiliative social interactions, but an acceptable alternative is to check the macaques are at least socially housed with the opportunity for normal social behaviour and there is no evidence of NHP induced injury. Our approach to scoring of staff-based indicators allows a degree of flexibility and rewards programmes which incorporate elements of good practice, though users can choose to focus on the other indicator types if they wish. Most of the animal-based indicators can be directly and objectively measured after only a short-period of staff training, and they do not overly disturb the animal. The information can be gathered during site inspections, daily observations, physical exams, and other activities, such as handling for scheduled scientific procedures. Where there is the option to conduct more detailed, extended behavioural observations (e.g. analysis of closed-circuit television [CCTV] recordings), we would encourage this as it will provide greater insight into an animal’s welfare state, especially if compared against a baseline measurement of normal behaviour for individual animals during their active phase and prior to any study ( Council on Animal Care, 2019). A pilot study is underway to assess the time commitment required for completion of assessments using the tool, for a variety of group and colony sizes. It is possible that emerging approaches for automated recording and analysis of NHP behaviour will help to reduce the time required in the future ( Rushen *et al*., 2012; Witham, 2018; Bain *et al*., 2021).

The multi-dimensional assessment should be performed by experienced staff, ideally with a knowledge of the individual animals, so that changes in welfare status can be more readily identified. The indicators included in the protocol do not require veterinary diagnostic expertise or specialist animal behaviour knowledge to be accurately recorded, but the involvement of such experts in implementing the welfare assessment tool is encouraged, particularly in the interpretation of the findings of assessments using this tool. A team approach, with good communication among those involved and periodic testing of inter-observer reliability, will help to ensure reliable assessments and consistent use of the tool ( Clingerman & Summers, 2012; Lambeth *et al*., 2013). Individual animal records can be combined to give an overview of the colony, which can be reviewed periodically or compared with data from other colonies. If the tool is used as part of daily health checks, then scores can be compared over time and a greater severity score assigned where there is repeated evidence of impaired welfare.

To facilitate use of the GEN-MAC protocol in practice, an Excel version is available to download. The file incorporates the formulae for calculating the welfare scores. We encourage users of the protocol to provide us with feedback, so that the tool can be enhanced; please email mark.prescott@nc3rs.org.uk. When reporting use or adaptation of GEN-MAC in the literature, please use the following citation:

Generalised macaque welfare assessment protocol (GEN-MAC) https://doi.org/10.25405/data.ncl.19106960. From: Prescott MJ, Leach MC, Truelove MA. Harmonisation of welfare indicators for macaques and marmosets used or bred for research. *F1000Research* 2022: X-X ( https://doi.org/10.12688/f1000research.109380.1).

## Results

### Demographics

A total of 73 participants took part in this survey ( Leach *et al*., 2022). Of these 73 respondents, 67 (92%) worked with macaque species (rhesus macaques, *Macaca mulatta,* and cynomolgus/long-tailed/crab-eating macaques, *M. fascicularis*): 34 (47%) in neuroscience, 12 (16%) in breeding, nine (12%) in toxicology, four (5%) in infectious disease research, and eight (11%) in other disciplines. Six respondents (8%) worked with common marmosets, *Callithrix jacchus:* four in infectious research, one in neuroscience, and one in breeding.

### Macaque respondents

*Round 1, Phase 1 – rating of top ten indices*

**Figure 3.**
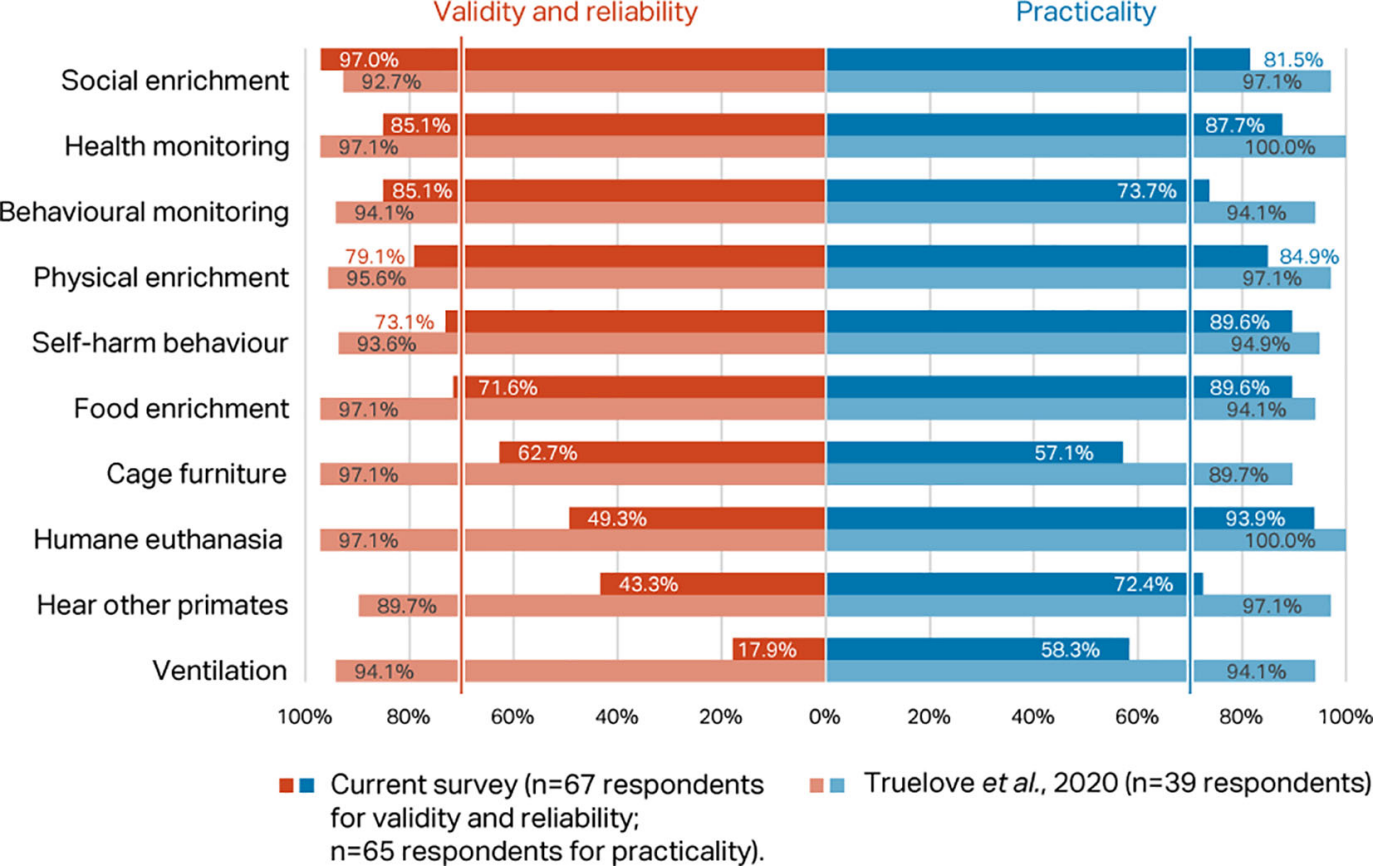
Comparison of validity and reliability and practicability scores (percentage of respondents) between the two Delphi consultations for macaques, showing the top ten indicators identified in Truelove *et al*. (2020).

Percentage scores for validity and reliability, and practicability, of the top ten macaque welfare indicators in the Truelove *et al*. (2020) Delphi were compared with the scores for the same indices in the current Delphi ( Figure 3). Considering respondents working with macaques only, there was agreement between the two consultations that presence of self-harm behaviour, provision of social, food, and physical enrichment, and health and behaviour monitoring are valid, reliable, and practicable welfare indicators for NHPs (>70% consensus). However, whilst included in the top ten indicators in Truelove *et al*. (2020), cage furniture, humane euthanasia, hear other NHPs, and room ventilation failed to reach the consensus criterion in the current survey.

*Round 1, Phase 2 – rating of 56 indices*

The percentage agreement scores for the more extensive list of macaque welfare indicators presented in Round 1, Phase 2 are given in Table 3. For Table 3 (macaques) and Table 5 (marmosets), “Practical” reflects two practicability categories that have been collapsed (practical + very practical). Those indices reaching less than 70% agreement for practicability have been shaded grey. The 56 indices have also been categorised into the following symbol-coded indicator types:

▪ **Animal-based**: behavioural ^#^, physiological/physical ^##^
▪ **Environment-based**: micro ^^^ (i.e. cage), macro ^^^^ (i.e. ambient)
▪ **Staff-based**: procedural and development ^+^, husbandry ^++^

**Table 3.** Ranking of validity and reliability and practicability scores for macaques, 56 indices (n=67 respondents *).

56 macaque indicators from Truelove *et al*. (2020)	Q3. Of the 56 indices, which do you think are the most valid and reliable for assessing NHP welfare?		Q4. How practical are the indices you selected for assessing NHP welfare?	
Potential indicator	Count (n)	Valid, reliable (%)	Count (n)	Practical (%)
Self-harm behaviour ^#^	54	80.6	44	81.5
Social enrichment ^^^	52	77.6	45	86.5
Cage dimensions ^^^	51	76.1	43	84.3
Body weight ^##^	49	73.1	40	81.6
Health monitoring programme ^++^	49	73.1	38	79.2
Appetite ^#^	47	70.1	35	74.5
Staff training ^++^	47	70.1	38	80.9
Positive reinforcement training ^++^	47	70.1	36	76.6
NHP induced injuries ^##^	46	68.7	38	84.4
Behavioural management programme ^++^	46	68.7	34	73.9
Vertical space ^^^	45	67.2	41	91.1
Cage furniture ^^^	43	64.2	40	93.0
Blood in faeces/urine ^##^	43	64.2	34	79.1
Huddled posture ^#^	43	64.2	38	88.4
Physical enrichment ^^^	43	64.2	40	93.0
Stereotypy ^#^	43	64.2	31	72.1
Social density ^^^	42	62.7	32	76.2
Food enrichment ^^^	41	61.2	38	92.7
See other NHPs ^^^	41	61.2	40	97.6
Mortality ^##^	40	59.7	31	79.5
Animal caregiver observations ^++^	40	59.7	35	87.5
Weaning age ^+^	40	59.7	29	72.5
Environmental complexity ^^^	39	58.2	29	74.4
Prolapse ^##^	38	56.7	30	78.9
Frequency of surgeries during lifetime ^+^	37	55.2	26	70.3
Humane euthanasia programme ^++^	36	53.7	31	86.1
Frequency of sedations during lifetime ^+^	36	53.7	26	72.2
Frequency of medical procedures during lifetime ^+^	36	53.7	26	72.2
Visual barriers between cages ^^^	34	50.7	31	91.2
Frequency of moves during lifetime ^+^	33	49.3	22	66.7
Visual barriers within cages ^^^	32	47.8	29	90.6
Discharges ^##^	32	47.8	28	90.3
Environmental-induced injuries ^##^	31	46.3	19	61.3
Rearing history ^+^	31	46.3	22	71.0
Provision of variety of food ^^^	30	44.8	25	83.3
Room temperature ^^ ^^	28	41.8	20	71.4
Field of view ^^^	28	41.8	15	55.6
Dyspnoea ^#^	26	38.8	19	73.1
Sensory enrichment ^^^	26	38.8	19	73.1
Fear of other NHPs ^#^	25	37.3	14	56.0
Disease surveillance ^++^	24	35.8	21	87.5
Destructible enrichment ^^^	24	35.8	22	91.7
Frequency of chair restraint ^+^	23	34.3	12	52.2
Light intensity ^^ ^^	22	32.8	21	95.5
Hear other NHPs ^^ ^^	22	32.8	19	86.4
Cage position ^^^	22	32.8	12	57.1
Room cleaning frequency ^++^	22	32.8	15	68.2
Manipulanda ^^^	21	31.3	15	71.4
Humidity ^^ ^^	20	29.9	15	78.9
Room ventilation ^^^^	18	26.9	14	77.8
Daily number of meals ^++^	18	26.9	14	77.8
Intentional exposure to novelty ^++^	18	26.9	9	50.0
See humans ^^^	17	25.4	15	88.2
Daily timing of meals ^++^	15	22.4	12	80.0
Frequency of inoculations during lifetime ^+^	12	17.9	9	75.0
Provision of browse ^^^	12	17.9	10	83.3

NHP=non-human primates.

* Fewer respondents replied to Q4.

Across the 56 welfare indicators presented to the macaque respondents (n=67), only eight (14.3%) met the *a priori* agreement level of ≥ 70% for validity and reliability; these were also considered practicable measures. Three were animal-based (self-harm behaviour, body weight, appetite), whilst the other five were environment- or staff-based (social enrichment, cage dimensions, health monitoring, staff training, positive reinforcement training). Three of these eight indices selected by respondents are in the top ten of Truelove *et al*. (2020) (self-harm behaviour, social enrichment, and health monitoring) (see *Extended data,* Supplementary Table 1 ( Leach *et al*., 2022)). Consensus for three additional indictors was nearly reached, with agreement levels between 65-69.99%; one was animal-based (NHP induced injuries) and the remainder were staff- and environment-based (behavioural management programme, vertical space).

Items were deemed more practicable to measure than they were valid and reliable, with 48 indicators (85.7%) meeting the threshold for consensus for practicability and two additional indicators approaching consensus (room cleaning frequency, frequency of moves during lifetime). Six indicators did not meet the consensus threshold for practicability when considering the hypothetical scenario; four of these were environment- or staff-based (field of view, intentional exposure to novelty, frequency of chair restraint, cage position) and two were animal-based (fear of other NHPs, environmentally induced injuries).

*Round 2 – measurement of selected welfare indicators*

**Figure 4.**
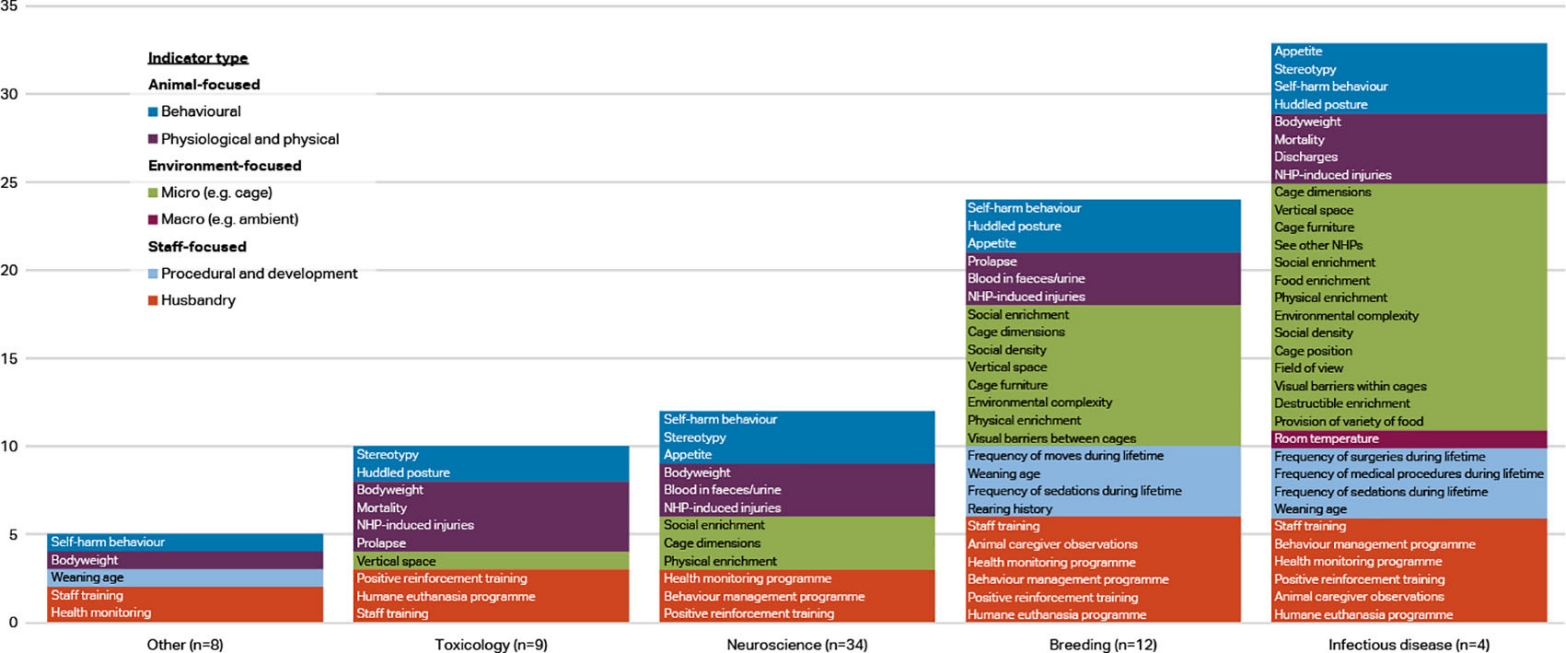
Indicators chosen as reliable and valid by two-thirds of respondents, macaques by discipline and indicator type (n=67 respondents).

Respondents working with macaques were classified into five categories: neuroscience (n=34), toxicology (n=9), infectious disease (n=4), breeding (n=12), and other (n=8). The ‘other’ category included the disciplines of reproduction, surgery, metabolic disease, and ethology. Figure 4 shows the indicators chosen as reliable and valid by two-thirds of macaque respondents (i.e. at or approaching consensus) by discipline and indicator type. Items approaching consensus (65-69.99%) are included in these results as some of the groups had small sample sizes and items would perhaps reach consensus with additional participants. Refer to *Extended data* Supplementary Table 2 for a complete list of the respondent agreement scores for validity and reliability of the indices by discipline category ( Leach *et al*., 2022).

More of the 56 welfare indices were considered valid and reliable by at least two-thirds of respondents in the infectious disease category (33 indices) and breeding category (24 indices), than in the other three categories. Whilst the number of animal-focused indices is relatively similar across the main disciplines (6-8 indices), the infectious disease and breeding categories also include procedural and developmental indices, as well as more micro-environment level indicators. Self-harm behaviour is one of the top four indices in all discipline categories but toxicology, and body weight appears as one of the top four as well in all except breeding. Provision of social enrichment appears in each discipline except toxicology and other. Potential explanations for the variation between the disciplines are given in the Discussion section.

Discipline groups discussed and reported how they would measure the potential welfare indicators deemed valid, reliable, and practicable. The top ten indices from the current survey and how they might be measured are given in Table 4. For each indicator, participants recommended that each should be measured at 91-100% of the population being assessed, whether the unit of measurement be at the individual or the facility level, to get a meaningful assessment. All indicators except staff training could be measured at the individual level, and there was a mix of methods for recording the indices, with observation, records, or both being recommended.

**Table 4.** Method of assessment, recording level, frequency, and duration for top ten indicators for macaques (n=8-10 groups across five disciplines).

Macaques	Recording level				Method		Notes	Duration and frequency
Indicator	Individual	Group/Cage	Room	Facility	Observation	Records		
Self-harm behaviour	*				O	R	Observation of behaviour and injury; record incidence and severity	One to multiple daily checks (up to 5 mins.)
Social enrichment	*	*			O	R	Presence/absence of programme; review of records	Daily or as needed (e.g. pair/group formation)
Cage dimensions	*			*	O		Per location	As required (i.e. caging change)
Body weight	*				O		Sedation may be needed	Daily to weekly
Health monitoring programme	*			*		R	Inspection; presence/absence programme	Semi-annually or annually; multiple daily checks
Appetite	*				O		Food consumed and feeding behaviour	Daily checks (1-10 mins. per cage)
Staff training				*		R	At personnel level, including competence	Ongoing or annually
Positive reinforcement training	*					R	Training logs (behaviour, frequency, duration, training stage, shaping plan)	At each session (daily to weekly)
NHP induced injuries	*				O		Visual inspection of the animal	One to multiple daily checks (as long as needed)
Behavioural management programme	*			*	O	R	Presence/absence of programme	Semi-annually or annually; multiple daily checks (2-5 mins. per animal)

NHP=non-human primates.

### Marmoset respondents

*Round 1, Phase 1 – rating of top ten indices*

**Figure 5.**
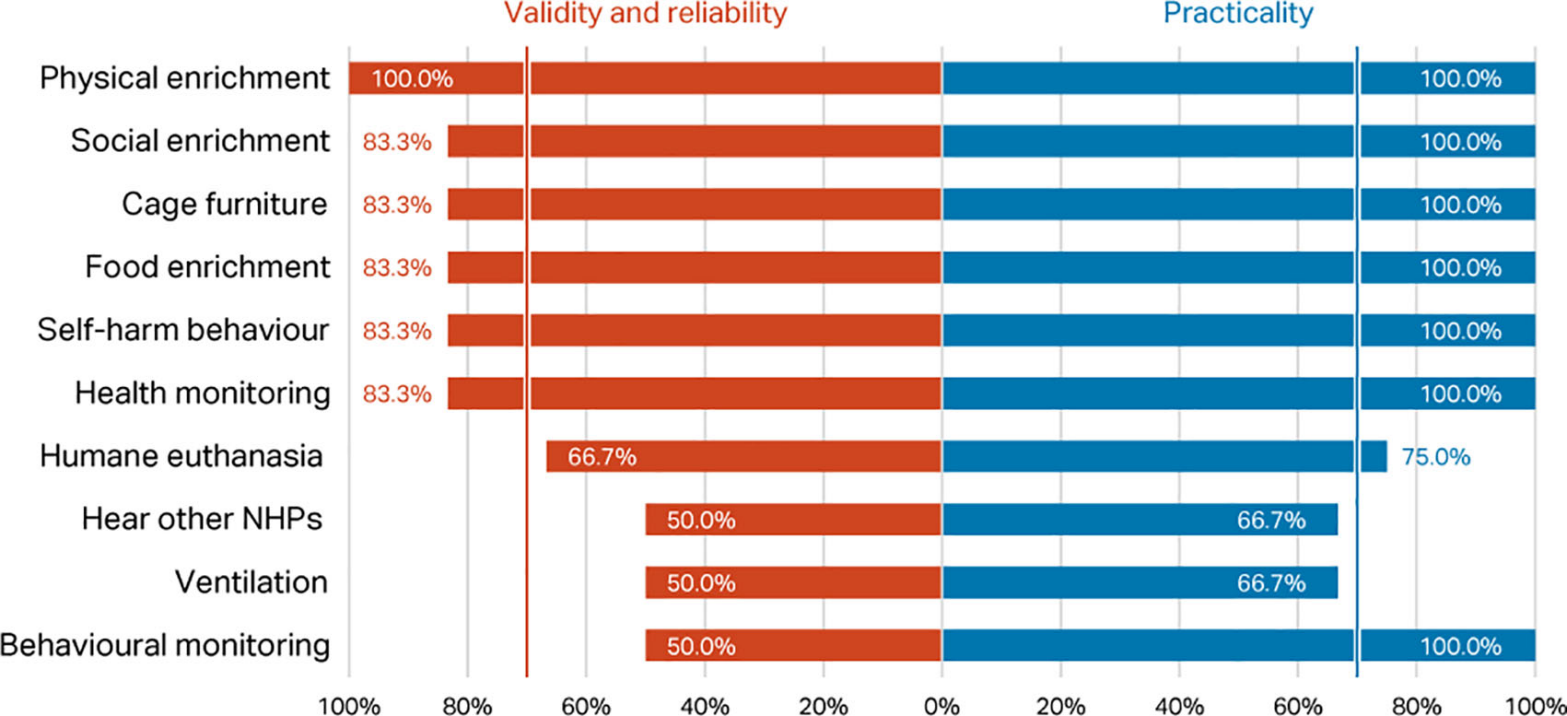
Validity and reliability and practicability scores (% of respondents) for marmosets, top ten indicators from Truelove *et al*. (2020) (n=6 respondents).

As was the case for macaque respondents, when presented with the top ten indices from Truelove *et al*. (2020), most respondents working with marmosets considered social, physical, and food enrichment to be indices that can be used to assess welfare, along with self-harm behaviours and the presence of a health monitoring programme; cage dimensions was also considered important. Hearing other NHPs and ventilation were not rated highly (selected by only 50% of respondents) ( Figure 5). A smaller proportion of respondents working with marmosets considered a behavioural management programme to be useful for assessing welfare (50%), than did those working with macaques (82.6%). Of these top ten indices, eight were considered practicable by all respondents, and all ten by at least two-thirds of respondents (potentially a consequence of the small sample size; n=6).

*Round 1, Phase 2 – rating of 56 indices*

Overall, 21 of the more extensive list of 56 indicators were considered valid and reliable measures for assessing marmoset welfare ( Table 5); eight met consensus, while the other 13 approached consensus. Of these 21, nine are animal-based, with the remainder comprised of environment-based (9) or staff-based (3) indicators. Of the 56 indicators, 50 (89.3%) were rated as practicable by at least two-thirds of the respondents working with marmosets.

**Table 5.** Ranking of validity and reliability and practicability scores for marmosets, 56 indies (n=6 respondents).

56 macaque indicators from Truelove *et al*. (2020)	Q3. Of the 56 indices, which do you think are the most valid and reliable for assessing NHP welfare?		Q4. How practical are the indices you selected for assessing NHP welfare?	
Potential indicator	Count (n)	Valid, reliable (%)	Count (n)	Practical (%)
Body weight ^##^	6	100	6	100.0
NHP induced injuries ^##^	6	100	5	83.3
Cage furniture ^^^	6	100	6	100.0
Huddled posture ^#^	5	83.3	5	100.0
Mortality ^##^	5	83.3	5	100.0
Blood in faeces/urine ^##^	5	83.3	5	100.0
Environmentally induced injuries ^##^	5	83.3	4	80.0
Physical enrichment ^^^	5	83.3	5	100.0
Self-harm behaviour ^#^	4	66.7	4	100.0
Stereotypy ^#^	4	66.7	4	100.0
Appetite ^#^	4	66.7	4	100.0
Food enrichment ^^^	4	66.7	4	100.0
Cage dimensions ^^^	4	66.7	3	75.0
Visual barriers within cages ^^^	4	66.7	4	100.0
Humidity ^^^^	4	66.7	3	75.0
Room temperature ^^^^	4	66.7	3	75.0
Room ventilation ^^^^	4	66.7	3	75.0
Light intensity ^^^^	4	66.7	3	75.0
Staff training ^++^	4	66.7	4	100.0
Animal caregiver observations ^++^	4	66.7	4	100.0
Disease surveillance ^++^	4	66.7	4	100.0
Discharges ^##^	3	50.0	3	100.0
Dyspnoea ^##^	3	50.0	3	100.0
Social enrichment ^^^	3	50.0	3	100.0
See other NHPs ^^^	3	50.0	3	100.0
See humans ^^^	3	50.0	3	100.0
Vertical space ^^^	3	50.0	3	100.0
Visual barriers between cages ^^^	3	50.0	3	100.0
Provision of variety of food ^^^	3	50.0	2	66.7
Hear other NHPs ^^^^	3	50.0	3	100.0
Health monitoring programme ^++^	3	50.0	2	66.7
Humane euthanasia programme ^++^	3	50.0	2	66.7
Daily number of meals ^++^	3	50.0	3	100.0
Daily timing of meals ^++^	3	50.0	3	100.0
Rearing history ^+^	3	50.0	2	66.7
Weaning age ^+^	3	50.0	2	66.7
Frequency of moves during lifetime ^+^	3	50.0	1	33.3
Frequency of sedations during lifetime ^+^	3	50.0	1	33.3
Frequency of surgeries during lifetime ^+^	3	50.0	2	66.7
Fear of other NHPs ^#^	2	33.3	2	100.0
Behavioural management programme ^++^	2	33.3	2	100.0
Positive reinforcement training ^++^	2	33.3	2	100.0
Frequency of inoculations during lifetime ^+^	2	33.3	1	50.0
Frequency of medical procedures during lifetime ^+^	2	33.3	1	50.0
Cage position ^^^	2	33.3	1	50.0
Field of view ^^^	2	33.3	2	100.0
Social density ^^^	2	33.3	2	100.0
Environmental complexity ^^^	2	33.3	2	100.0
Prolapse ^##^	1	16.7	1	100.0
Sensory enrichment ^^^	1	16.7	1	100.0
Destructible enrichment ^^^	1	16.7	1	100.0
Provision of browse ^^^	1	16.7	1	100.0
Manipulanda ^^^	1	16.7	1	100.0
Intentional exposure to novelty ^++^	1	16.7	1	100.0
Room cleaning frequency ^++^	1	16.7	1	100.0
Frequency of chair restraint ^+^	0	0.0	0	0.0

NHP=non-human primates.

**Figure 6.**
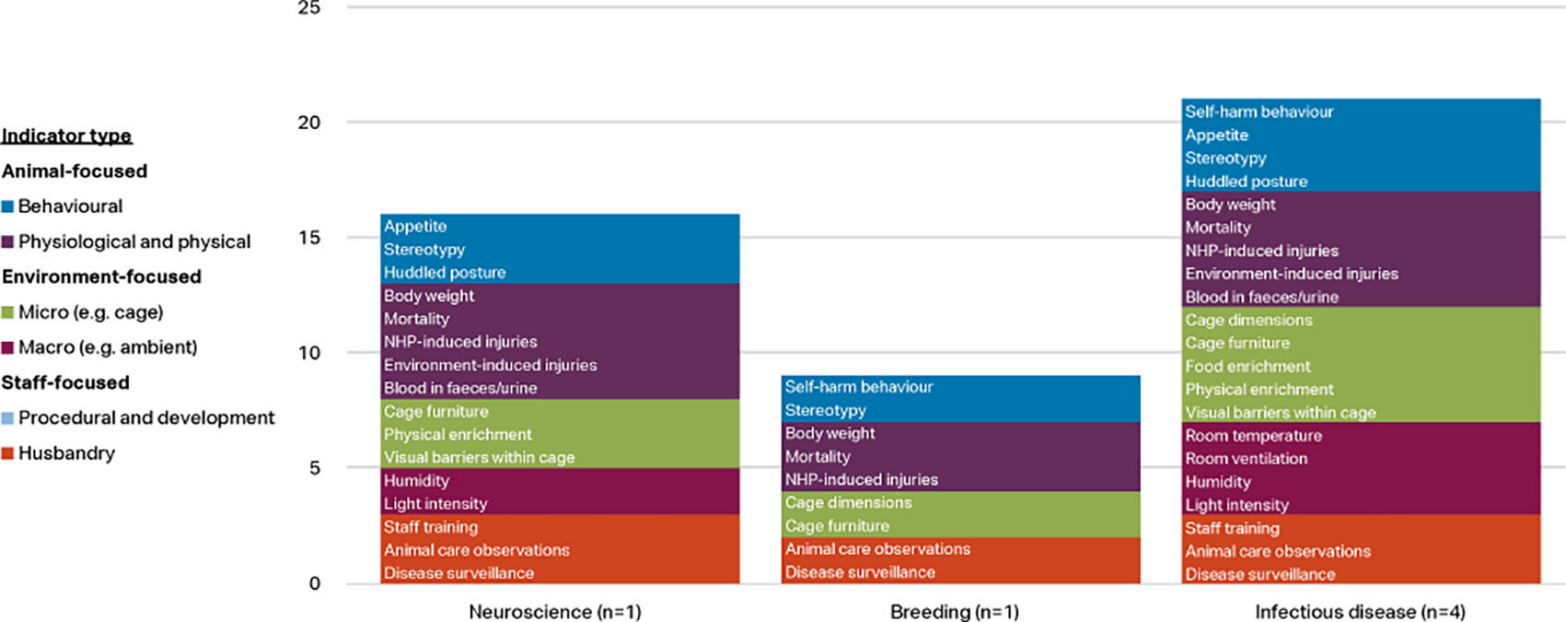
Indicators chosen as reliable and valid by two-thirds of respondents, marmosets by discipline and indicator type (n=6 respondents).

The few respondents working with marmosets were classified into three categories: infectious disease (n=4), neuroscience (1) and breeding (n=1). Indicators chosen as reliable and valid by at least two-thirds of these respondents are shown by discipline and indicator type in Figure 6. Items approaching consensus (65-69.99%) are included in these results as the groups had very small sample sizes and items would perhaps reach consensus with additional participants. More of the 56 welfare indices were considered valid and reliable by at least 70% of respondents in the infectious disease category (21 indices) and neuroscience category (16 indices), than in breeding (9). Seven indices were selected in all three disciplines (stereotypy, body weight, mortality, NHP induced injuries, cage furniture, animal care observations, and disease surveillance) and all 16 indices in the neuroscience category were also selected by the infectious disease group.

*Round 2 – measurement of selected welfare indicators*

The marmoset experts also discussed and reported how they would measure the potential welfare indices deemed valid, reliable, and practicable. The top eight indices from the current survey and how they might be measured for this species are given in Table 6. For each indicator, participants recommended that each should be measured at 91-100% of the population being assessed, whether the unit of measurement be at the individual or the facility level, to get a meaningful assessment. All indicators could be measured at either the individual or cage level, as appropriate, with NHP induced injuries also being assessed at the room level. There was a mix of methods for recording these indices, with observation, records, or both being recommended.

**Table 6.** Method of assessment, recording level, frequency, and duration for top eight indicators for marmosets (n=3 discipline groups).

Marmosets	Recording level				Method		Notes	Duration and frequency
Indicator	Individual	Group/Cage	Room	Facility	Observation	Records		
Body weight	*				O	R	Weight and body condition score	Regular health checks, or when handling animal for experiment or management needs
NHP induced injuries	*		*		O			As needed (up to 10 minutes) over a year period (for comparison)
Cage furniture		*				R		Housing records
Huddled posture	*				O			At each physical check (60s)
Mortality	*					R		Intermittently as needed
Blood in faeces/urine		*			O			Daily checks (up to 1 minute)
Environmentally induced injuries	*				O			As needed (up to 5 minutes) over a year period (for comparison)
Physical enrichment		*			O	R	Observation of use and records of what is provided.	As needed if injuries occur or items not used.

NHP=non-human primates.

*Macaque and marmoset respondents*

Considering both macaque and marmoset respondents (N=73), the whole group’s level of disagreement about the validity and reliability of the top ten indicators identified in Truelove *et al*. (2020) was high in both phases (Phase 1, α=0.1993; Phase 2, α=0.0915); however, levels remained relatively consistent between phases (Δ 0.1078), indicating group stability. The movement that occurred between phases was in the direction of disagreement (signifying divergence). Likely, this was a result of the increased options provided to the respondents between phases (i.e., more potential indices in Phase 2) and not requiring ranking of the same ten items across each phase; respondent fatigue is also a possibility. When asked to indicate which of 56 potential macaque welfare indicators are valid and reliable (Q3) for assessing NHP welfare, those ten initially presented (Q1) shifted in importance as evidenced by the proportion of respondents who selected an item as important for assessing welfare ( Table 7). Across the two phases, the respondents’ average inter-rater agreement was 78%; however, there were 12 respondents who had scores below 70%.

**Table 7.** Respondent percentage agreement score shift between Phases 1 and 2 of Round 1 for Truelove *et al*. (2020) top ten indicators.

	Humane euthanasia	Health Monitoring	Food enrichment	Physical enrichment	Social enrichment	Self-harm behaviour	Ventilation	Behavioural management	Hear other NHPs	Cage furniture
Percent of respondents selecting indicator, Q1 (Phase 1)	50.7	84.9	72.6	80.8	95.9	74.0	20.5	82.2	43.8	64.4
Percent of respondents selecting indicator, Q3 (Phase 2)	53.4	71.2	61.6	65.8	75.3	79.5	30.1	65.8	34.2	67.1
Percent change between Phases 1 and 2 in Round 1	2.7	-13.7	-11.0	-15.1	-20.5	5.5	9.6	-16.4	-9.6	2.7

Across the two Delphi consultations, four indicators were identified as important for both marmosets and macaques: body weight, NHP induced injuries, physical enrichment, and cage furniture ( Table 8).

**Table 8.** Summary of top indicators, comparing species and Delphi consultations.

Delphi consultation	Self-harm behaviour	Social enrichment	Cage dimensions	Body weight	Health monitoring	Appetite	Staff training	Positive reinforcement training	NHP induced injuries	Behavioural management	Humane euthanasia	Food enrichment	Physical enrichment	Ventilation	Hear other primates	Cage furniture	Huddled posture	Mortality	Environmentally induced injuries	Blood in faeces/urine
Macaques, top ten, Truelove *et al*. (2020) (n=39)	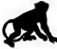	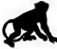			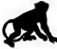					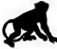	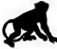	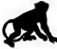	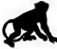	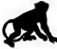	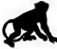	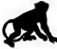				
Macaques, top ten, Current survey (n=67) *	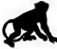	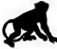	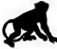	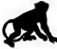	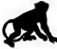	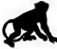	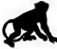	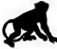	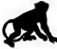	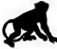										
Marmosets, top eight, Current survey (n=6)				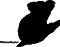					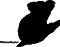				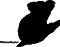			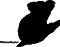	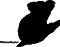	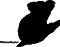	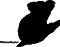	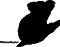

NHP=non-human primates.

* Top eight are valid, reliable, and practicable; NHP induced injuries and behavioural management are valid and reliable.

## Discussion

This study aimed to achieve consensus on effective indices of welfare for macaques and marmosets bred and used for research through expert consultation about the validity, reliability and practicability of a range of potential indicators. It builds upon the previous Delphi consultation of Truelove *et al*. (2020) by surveying a larger population of macaque experts working within a broader range of countries (predominantly within the EU) and collecting information on how top-ranking indices should be measured. The larger population also enabled us to explore differences between disciplines (though sample sizes were small for some discipline categories). By combining data from the two consultations, we were able to develop a generalised protocol for welfare assessment of macaque species.

We chose a Delphi process owing to the ability to survey a large number of experts anonymously and independently, without the opinions of any one respondent/group dominating the discussion, and to provide controlled feedback, helping to reduce noise and converge upon quality indicators. The systematic Delphi approach is more rigorous than other group consensus approaches, like case studies or focus groups ( Boulkedid *et al.,* 2011). However, it does have limitations which impact on our interpretation of the results (outlined below).

Considering first respondents working with common marmosets, of the 56 indicators presented in Round 1 Phase 2, only 37.5% (21/56) were considered valid and reliable for assessing marmoset welfare, and 89.3% (50/56) were rated as practicable by at least two-thirds of the respondents. This is not surprising given these indicators were those furnished from the macaque literature and experts in Truelove *et al*. (2020). Of the six indicators rated as not practicable, five were staff-based and one was a micro-environment indicator. Of note in terms of species differences is the observation that ambient environment indicators such as humidity, room temperature, room ventilation, and light intensity were considered valid and reliable by two-thirds of marmoset respondents but not so of macaque respondents, probably reflecting the physical needs of these tropical New World monkeys, which are different to those of macaques and temperate living humans ( Buchanan-Smith, 2010b).

Indicators ranked important for assessing the welfare of common marmosets were body weight, NHP and environmentally induced injuries, cage furniture, huddled posture, mortality, blood in excreta, and physical enrichment. These findings should be viewed as preliminary given only six of our 73 respondents (8%) worked with this species and the indices were from Truelove *et al*. (2020). Nonetheless, whilst they cannot be said to be indicative of the marmoset research community, these findings have value in identifying potential effective indicators that can be further explored in a subsequent Delphi process involving a larger population of subject experts. We consider it important to conduct this exercise, given the resurgence in the use of this species in biomedical research ( Colman *et al*., 2021). Some of the chosen indicators may reflect signs typically associated with marmoset wasting syndrome, a disease which causes morbidity and mortality in captive colonies ( Ludlage & Masfield, 2003). We note also there was some inter-species application, as indicated by the percentage agreement scores of marmoset experts for seven of the top ten macaque indices in Truelove *et al*. (2020). It is valuable to have identified welfare assessment indicators that could be applied to multiple NHP species, particularly when conducting a limited-time, on-site assessment.

When presented with 115 potential indicators for macaques, participants in the Truelove *et al*. (2020) Delphi selected 56 of these as valid, reliable, and practicable within the context of a hypothetical scenario involving 500 animals; environment-based and staff-based indicators (44) were selected more than three times animal-based indicators (12). In the current study and with the same scenario, of the 56 indices, only eight were found to be valid, reliable, and practicable by at least 70% agreement of the macaque respondents. Three of the eight were animal-based (self-harm behaviour, body weight, appetite); the remainder were either environment-based (social enrichment, cage dimensions) or staff-based (health monitoring programme, staff training, positive reinforcement training). In addition, NHP induced injuries (animal-based) and presence of a behavioural management programme (staff-based) approached consensus at 68.7%.

It is notable that no physiological indicators and only one behavioural indicator (self-harm behaviour) are included in the top ten of Truelove *et al*. (2020), probably reflecting the greater effort required in collecting animal-based data to assess welfare (though 12 animal-based indicators, including body weight, appetite, and NHP induced injuries did reach consensus in Truelove *et al*., 2020). We speculate that the predominantly European participants of the current Delphi were more open to animal-based indicators than the predominantly North American participants in Truelove *et al*. (2020) because European macaque colonies tend to be smaller and there is perhaps more staff resource available to obtain information requiring direct measurement. Environment- and staff-based indicators can generally be assessed with more immediacy and ease, and without specialist equipment or judgement (e.g. whether cage furniture is present in the enclosure). However, it should be noted that the mere presence of something does not give a full picture of its contribution to NHP welfare; the quality of the item, and how much it is used, and by which animals, are also important factors.

Of the eight indicators selected in Phase 2, three of these also appeared in the top ten of Truelove *et al*. (2020) presented in Phase 1 (self-harm behaviour, social enrichment, health monitoring programme), strongly suggesting that these indicators are considered critical for the assessment of macaque welfare. Also exceeding or approaching the 70% threshold in Phase 1 were a behavioural management programme, physical enrichment, and food enrichment, suggesting their consideration as well. Social enrichment was rated the top indicator in Phase 1 (>94% of respondents), reflecting the importance of companionship for psychological well-being in these animals. Social enrichment and self-harm behaviour are well known as important indicators of good and poor welfare, respectively, in NHPs, and there is a large literature on their incidence and relevance to macaque well-being, so it is not surprising that there was consensus agreement on their importance in both Delphi consultations. Fewer than two-thirds of respondents felt a humane euthanasia programme, hearing other NHPs, cage furniture, and ventilation could be used to assess welfare. Opinion was split on how practical cage furniture and ventilation are as welfare indices for macaques. Agreement scores for practicability were generally higher in Truelove *et al*. (2020) than in the current study, possibly reflecting differences in expert demographics, sample size, and methodology.

A greater number of the 56 indicators were considered valid and reliable by respondents working with macaques in infectious disease research and breeding, than in neuroscience, toxicology and other disciplines, perhaps reflecting a greater awareness of the impact of surgical and husbandry procedures, and the cage environment, on the welfare of these animals ( Figure 4). Macaques in neuroscience also undergo frequent sedations, surgeries, and medical procedures, so it is curious these did not approach consensus in this category but did so in infectious disease. Within neuroscience, body weight and appetite are included in the top four indices, reflecting that many macaques used in neuroscience undergo food or fluid control to motivate them to work on behavioural or cognitive tasks whilst brain activity is measured. Self-harm behaviour and stereotypies are within the top seven, reflecting the practice of monitoring between experimental manipulations behavioural changes which could compromise the validity of the NHP model. Although they did not meet the set threshold for inclusion as items to rate by all participants, additional indicators suggested by respondents within this discipline included activity level (including the presence of depressive-like behaviour or non-alert inactive behaviour), engagement with and performance on experimental tasks, and water intake (again reflecting the scientific procedures involved).

Within infectious disease, more than half of the indicators (33/56) were considered valid and reliable by three-quarters of respondents, with over 40% (14/33) of these being cage-based. Appetite, body weight, and mortality are in the top five, reflecting that many of the macaques used in such studies will experience disease ( Prescott *et al*., 2021). Additionally, to round out the top five are stereotypy and self-harm behaviour. Similar to neuroscience, these indices reflect the practice of monitoring behavioural changes between experimental manipulations over prolonged periods of time.

Ten indicators were selected by more than two-thirds of respondents in toxicology, drawn from a range of categories – behavioural, physiological, husbandry-based, and cage-based. Body weight and mortality checks are routinely performed as part of regulatory toxicology studies. Most animals are euthanised for pathology when assigned to toxicology studies, which might account for the appearance of a humane euthanasia programme in the top four. Huddled posture may reflect sickness due to test drug administration.

Within breeding, 24 of the 56 indices (42.9%) were selected by more than two-thirds of respondents. Of the top ten, five are cage-based and four husbandry-based, reflecting the large group sizes and number of animals to be monitored in breeding units, as well as the relative lack of need for scientific procedures and for welfare data for a scientific purpose. The inclusion of indicators such as social density, vertical space, environmental complexity, cage furniture, and visual barriers probably reflects the more spacious environments often afforded to breeding animals.

That some indices are contextualized differently across specializations is not surprising. For example, those who require chair restraint for handling of monkeys to do their research might find frequency of such restraint to be a more useful indicator than those who do not. This brings to light the difficulty in assessing animal welfare and the complexities of using indicators – they must be well defined, validated for the species for which they are applied, and need be not only practical to measure but also reliable across time and raters. Agreement on which of these is most important is coloured by culture and work experience as well as discipline perspective ( Duijvesteijn *et al*., 2014), and when working as a group, the results of the process will also be subject to the composition of the panel. In the current study, the majority of respondents working with macaques did so in neuroscience, and most respondents were primarily from the UK and EU, whereas those in the Truelove *et al*. (2020) study were primarily from the USA. Differences exist between these regions in how laboratory NHPs are housed and managed, partly due to the oversight regulations. For example, minimum cage space requirements for NHPs are considerably smaller in the USA than in the UK and EU member countries. Under Directive 2010/63/EU ( European Union, 2010), the minimum volume for macaques from three years of age is 1.8m ^3^ /64ft ^3^ per animal, reflecting the value placed on providing housing that allows for exercise and the expression of ethologically relevant behaviours, such as running, climbing, leaping and hiding from companions ( NC3Rs, 2017). Under the ILAR Guide ( National Research Council, 2011), the minimum volume per macaque up to 10kg is 0.25m ^3^/9ft ^3^, and this space allocation was not increased in the 2011 revision. One possible reason for this disparity in minimum cage space is that UK and EU NHP facilities tend to house considerably fewer animals than those in the USA. Irrespective of these regional differences, our two Delphi consultations have been able to identify critically important indices for macaque welfare.

## Conclusions

We have identified context appropriate indicators that are valid, reliable, and practicable for assessing the welfare of macaques and marmosets bred and used for research, including in toxicology, neuroscience, and infectious disease, potentially benefiting far in excess of 100,000 NHPs used globally per year by improving welfare assessment, minimisation of harm and evaluation of the impact of refinement techniques. In ranking potential welfare indicators, we have identified those indicators considered the most important by experts and narrowed the field for further investigation and validation of both species-specific and general indicators. We have used the top-ranking indicators for macaques identified by experts in our two Delphi consultations, and agreement on how these should be measured, to develop a practical and generalised welfare assessment protocol to support laboratory staff in monitoring and optimising macaque health and well-being (GEN-MAC). Our work to harmonise welfare indicators and assessment should facilitate inter-lab comparative studies, data-sharing to boost sample sizes in research asking welfare-focused questions, and benchmarking of welfare standards between facilities. It would be good to build upon this momentum and achieve further consensus and harmonisation globally, involving Pacific Rim countries in addition to North America and Europe. Further validation of the proto-protocol, and of the top-ranking welfare indicators, would also be welcome. Funding opportunities are available from the NC3Rs, other bioscience organisations, and animal welfare charities.

## Data availability

### Underlying data

Newcastle University research repository: Survey Data and Supplementary Tables. https://doi.org/10.25405/data.ncl.19106960 ( Leach *et al*., 2022).

This project contains the following underlying data:

▪ Survey 1 Data anonymised.xlsx (Anonymised data set generated during Survey 1)
▪ Survey 2 Data anonymised.xlsx (Anonymised data set generated during Survey 2)

### Extended data

Newcastle University research repository: Survey Data and Supplementary Tables. https://doi.org/10.25405/data.ncl.19106960 ( Leach *et al*., 2022).

This project contains the following extended data:

▪ Supplementary Table 1.doc (Comparison of validity and reliability and practicability respondent scores between two Delphi exercises, macaques, 56 welfare indices)
▪ Supplementary Table 2.doc (Macaque respondent agreement scores for validity and reliability by discipline, 56 welfare indices, n=67 respondents)

Data are available under the terms of the Creative Commons Zero “No rights reserved” data waiver (CC0 1.0 Public domain dedication).
