# Supplementary material for: Harmonisation of welfare indicators for macaques and marmosets used or bred for research
Source: F1000Res. 2023 May 22;11:272. Originally published 2022 Mar 3. [Version 3] doi: 10.12688/f1000research.109380.3 (PMC9459172; doi:10.12688/f1000research.109380.3)
Supplement: Peer review history

# Reviewer report: Reviewer response for version 3

**DOI:** 10.5256/f1000research.145912.r174510

**Published:** 2023-06-01

**Authors:** Kristine Coleman

**Affiliations:** 1 Division of Comparative Medicine, Oregon National Primate Research Center, Oregon Health and Science University, Beaverton, OR, USA

The authors have done a nice job on this revision.

Are the 3Rs implications of the work described accurately?

Yes

Is the work clearly and accurately presented and does it cite the current literature?

Yes

If applicable, is the statistical analysis and its interpretation appropriate?

Yes

Are all the source data underlying the results available to ensure full reproducibility?

Yes

Is the study design appropriate and is the work technically sound?

Yes

Are the conclusions drawn adequately supported by the results?

Yes

Are a suitable application and appropriate end-users identified?

Yes

Are sufficient details of methods and analysis provided to allow replication by others?

Yes

Reviewer Expertise:

Behavioral management

I confirm that I have read this submission and believe that I have an appropriate level of expertise to confirm that it is of an acceptable scientific standard.

# Reviewer report: Reviewer response for version 2

**DOI:** 10.5256/f1000research.123055.r148327

**Published:** 2022-09-13

**Authors:** Kristine Coleman

**Affiliations:** 1 Division of Comparative Medicine, Oregon National Primate Research Center, Oregon Health and Science University, Beaverton, OR, USA

This MS offers an interesting approach to assessing welfare. It is well written and provides important information. 

 My main comment concerns the inclusion of marmosets. There were only 6 people who participated in the marmoset Delphi consultation, and those questions were based on a macaque welfare assessment. The authors mention that they were not able to generate a welfare protocol for marmosets due to these limitations (which sort of contradicts the title a bit). I am not sure what including marmosets really adds to this MS. The authors might consider re-focusing on macaques, or explain why including marmosets given these limitations is important. 

 I have a few additional, minor comments. 

**Introduction**

 It might be helpful if the authors briefly describe the Delphi consultation when they first bring it up, as opposed to at the end of the introduction.

 Second to last paragraph: The first sentence of this paragraph mentions macaque welfare, so it isn't necessarily clear why marmosets were addressed as well. The authors might consider changing "macaque" to "NHP" in that sentence. Also, it is not necessarily clear why the macaque assessment information (i.e., from Truelove et al 2020) was used as a basis for marmoset welfare. 

**Methods**

 It might be helpful to provide the percent of participants who were researchers (PhD level?), veterinarians, and animal techs, as well as those working with macaques vs marmosets, etc.

Are the 3Rs implications of the work described accurately?

Yes

Is the work clearly and accurately presented and does it cite the current literature?

Yes

If applicable, is the statistical analysis and its interpretation appropriate?

Yes

Are all the source data underlying the results available to ensure full reproducibility?

Yes

Is the study design appropriate and is the work technically sound?

Yes

Are the conclusions drawn adequately supported by the results?

Yes

Are a suitable application and appropriate end-users identified?

Yes

Are sufficient details of methods and analysis provided to allow replication by others?

Yes

Reviewer Expertise:

Behavioral management

I confirm that I have read this submission and believe that I have an appropriate level of expertise to confirm that it is of an acceptable scientific standard, however I have significant reservations, as outlined above.

# Response

**Published:** 2023-03-20

**Authors:** Melissa TRUELOVE

**Affiliations:** YNPRC/Emory Univ, USA

We thank the reviewer for her comments on the manuscript and recommendations for improvement. 

 Regarding the reviewer's comment concerning the inclusion of marmosets, we were obligated to publish the full data set from the Delphi consultation, therefore included the data on welfare indicators considered reliable, valid and practicable by laboratory staff working with *Callithrix jacchus*. In the manuscript, we have been careful to acknowledge the small sample size for the marmoset data, and we state that the marmoset findings should be viewed as preliminary. Inclusion of both species’ data allow for comparisons to be made and emphasizes the importance of doing such work per species. We have recommended that a subsequent Delphi process involving a larger population of subject experts further explore welfare indicators specific to marmosets, especially given their increasing use in research.

 At the reviewer's suggestion we have added additional text in the introduction to briefly describe the Delphi consultation when it is first mentioned.

 Regarding the second to last paragraph in the introduction, "macaque” was used in the first sentence to refer to the development of a protocol for this genus rather than for NHPs in general. In this paragraph, a sentence has been added for clarity to address why macaque assessment information was used as a basis for marmoset welfare: " *Macaca *experts either rejected or accepted the indices as measures of welfare for macaques as did *Callithrix* experts for marmosets, revealing whether welfare indicators identified for once species are applicable to other NHP species used or bred for research."

 Data pertaining to respondent profession were not captured on the survey so are not available for reporting in the Methods section. In the Results section, the percent of respondents working with each species and in what context (eg, neuroscience, breeding) is reported.

 We thank the reviewer again for her constructive feedback.

# Reviewer report: Reviewer response for version 2

**DOI:** 10.5256/f1000research.123055.r148326

**Published:** 2022-09-02

**Authors:** Jan A. M. Langermans

**Affiliations:** 1 Animal Science Department, Biomedical Primate Research Centre (BPRC), Rijswijk, The Netherlands; 2 Population Health Sciences, Animals In Science and Society, Veterinary Faculty, Utrecht University, Utrecht, The Netherlands

Welfare of animals used for research purposes is an important and essential topic. This is even more the case when this involves non-human primates. Welfare assessment tools should be based on objective measures and must be both thorough and practical to make it work and make it acceptable for the research community.

 In this paper, Prescott et al build upon welfare indicators identified in an earlier study by Truelove that was performed in North America. In this study a  Delphi consultation was done with a group of NHP experts using 56 indicators from the earlier study. The aim was to achieve consensus for NHP welfare indicators. The methods and data are clearly described. From the results a group of clear indicators that are both practical and reliable could be identified. This is an important addition to the toolbox of welfare assessment of NHP. My only proble is that the authors claim this for both macaques and for marmosets.

 Unfortunately, the group of marmoset users was also in this study very limited (also in the earlier study). Although still important to mention, it might be a bit premature to conclude that these indicators are generally applicable to assess marmoset welfare. I agree that a number of indicators can be applicable to both marmosets and macaques, but there are certainly also marmoset specific indicators. Given the limited number of marmoset users included in this study, I am a bit sceptic  about including "marmosets" in the title. However, the over-all data are importnat and helpful for welfare assessment in general by the NHP research community and I strongly recommend indexing of this paper.

Is the work clearly and accurately presented and does it cite the current literature?

Yes

If applicable, is the statistical analysis and its interpretation appropriate?

I cannot comment. A qualified statistician is required.

Are all the source data underlying the results available to ensure full reproducibility?

Yes

Is the study design appropriate and is the work technically sound?

Yes

Are the conclusions drawn adequately supported by the results?

Partly

Are sufficient details of methods and analysis provided to allow replication by others?

Yes

Reviewer Expertise:

animal welfare specialist with expertise in NHP

I confirm that I have read this submission and believe that I have an appropriate level of expertise to confirm that it is of an acceptable scientific standard.

# Response

**Published:** 2022-09-12

**Authors:** Mark Prescott

**Affiliations:** National Centre for the Replacement, Reduction and Refinement of Animals in Research, UK

We thank the reviewer for his comments on the manuscript. We are pleased the importance of this work for improving non-human primate welfare has been recognised.

 Marmosets are included alongside macaques in the title of the paper because we present data on welfare indicators considered reliable, valid and practical by laboratory staff working with *Callithrix jacchus*. It was important for us to publish the full data set from the Delphi consultation.

 However, we have been careful in the paper to acknowledge the small sample size for the marmoset data, and we state that the marmoset findings should be viewed as preliminary. We have recommended that a subsequent Delphi process involving a larger population of subject experts further explore welfare indicators for marmosets, especially given their increasing use.

# Reviewer report: Reviewer response for version 2

**DOI:** 10.5256/f1000research.123055.r144282

**Published:** 2022-07-21

**Authors:** Augusto Vitale

**Affiliations:** 1 Center for Behavioural Science and Mental Health, Istituto Superiore di Sanità (ISS), Rome, Italy

I am convinced by the corrections added to the paper by the authors, and by their responses to my comments.

 In my opinion now the article is suited for Indexing.

Are the 3Rs implications of the work described accurately?

Yes

Is the work clearly and accurately presented and does it cite the current literature?

Yes

If applicable, is the statistical analysis and its interpretation appropriate?

I cannot comment. A qualified statistician is required.

Are all the source data underlying the results available to ensure full reproducibility?

Yes

Is the study design appropriate and is the work technically sound?

Partly

Are the conclusions drawn adequately supported by the results?

Partly

Are a suitable application and appropriate end-users identified?

Yes

Are sufficient details of methods and analysis provided to allow replication by others?

Yes

Reviewer Expertise:

animal behaviour, animal welfare, primatology, ethics of research

I confirm that I have read this submission and believe that I have an appropriate level of expertise to confirm that it is of an acceptable scientific standard.

# Reviewer report: Reviewer response for version 1

**DOI:** 10.5256/f1000research.120873.r126226

**Published:** 2022-03-22

**Authors:** Augusto Vitale

**Affiliations:** 1 Center for Behavioural Science and Mental Health, Istituto Superiore di Sanità (ISS), Rome, Italy

The aim of this paper is to identify shared indicators for the level of welfare of macaques and marmosets in research laboratories, using a Delphi consultation during a NC3Rs meeting on primate welfare. The results pointed out at some reliable indicators for both species, with some interesting differences: self-harm behaviour and social enrichment were chosen as being important for macaques, whereas body weight for marmosets.

 I found the paper very informative and very relevant. These are the kind of studies which are really needed to improve the conditions of NHP in captivity, so I really applaud the authors' efforts. However, I have some concern about part of the article's content. In particular, although it is very informative and important for the macaques, less so for the marmosets. I don't feel that the authors have enough responses from the marmoset community to draw conclusions. There is in this paper a real unbalance when it comes to deal with the two species. I understand that this is a big ask, but I would feel more comfortable with this paper if the marmosets would be left to develop in another time, with more data available.

 In particular:

 In the "Research highlights" table, under "Scientific benefit(s)" and "Current applications" marmosets are not mentioned, supporting my general impression.

 Table 2: Some of the scoring criteria appear to be "states" (e.g. presence of injuries"), other "events" (e.g. affiliative behaviours). Is there any possibility to have an idea on how these observations would be carried out, in terms of timing, frequency...? 

 In the section "Results - Demographics" we have 67 colleagues working with macaques, and 6 working with marmosets. It appears to me greatly unbalanced. I would then delete the responses from the marmosets' people, for later wider participation.

 Table 6 reports the results for marmosets, however it is based on a very limited sample size. For example, just one person replied in the area of neuroscience - I would suggest to delete it.

 In the "Conclusions" section, there is no mention of the results on marmosets.

 Therefore, I recommend the approval of the present paper, but I consider it a "macaque paper", so:

 i) if the authors delete any reference to the marmoset survey;

 ii) or they play down very much that part, perhaps mentioning the results in the final discussion, underlying the necessity and need to compare different species, looking at their preliminary data on marmosets.

Are the 3Rs implications of the work described accurately?

Yes

Is the work clearly and accurately presented and does it cite the current literature?

Yes

If applicable, is the statistical analysis and its interpretation appropriate?

I cannot comment. A qualified statistician is required.

Are all the source data underlying the results available to ensure full reproducibility?

Yes

Is the study design appropriate and is the work technically sound?

Partly

Are the conclusions drawn adequately supported by the results?

Partly

Are a suitable application and appropriate end-users identified?

Yes

Are sufficient details of methods and analysis provided to allow replication by others?

Yes

Reviewer Expertise:

animal behaviour, animal welfare, primatology, ethics of research

I confirm that I have read this submission and believe that I have an appropriate level of expertise to confirm that it is of an acceptable scientific standard, however I have significant reservations, as outlined above.

# Response

**Published:** 2022-03-30

**Authors:** Mark Prescott

**Affiliations:** National Centre for the Replacement, Reduction and Refinement of Animals in Research, UK

We thank the reviewer for his comments on the manuscript. We are pleased the importance of this work for improving non-human primate welfare has been recognised. The reviewer's comments focus on the marmoset data.

 We appreciate the sample size for respondents using common marmosets is small and had hoped that this would be larger at the 2019 Primate Welfare Meeting. It is important to note there was no separate marmoset survey.

 We considered not including the marmoset data in the paper, but decided it was important to do so for a variety of reasons:

1. We are obligated to publish the data provided by all of the participants of the 2019 Delphi consultation.
2. Publishing the full data (and making the data set available) is the most transparent approach, in line with contemporary good practice.
3. Including the marmoset and macaque findings allows for comparisons to be made between species/genera, which shows some interesting differences as the reviewer acknowledges.
4. This emphasises the importance of doing such work per species.

We would therefore like to retain the marmoset data within the paper.

 We have already been careful to acknowledge the small sample size for marmosets in the Discussion section (which, as the reviewer has recognised, is also reflected in the Research Highlights section):

*These findings should be viewed as preliminary given only six of our 73 respondents (8%) worked with this species and the indices were from Truelove et al. (2020). Nonetheless, whilst they cannot be said to be indicative of the marmoset research community, these findings have value in identifying potential effective indicators that can be further explored in a subsequent Delphi process involving a larger population of subject experts. We consider it important to conduct this exercise, given the resurgence in the use of this species in biomedical research (Colman et al., 2021).*

 We believe this text addresses the reviewer’s suggestions to mention the marmoset results in the final Discussion, consider the marmoset data preliminary, and to gather data from a larger population of marmoset experts. We also comment in the Discussion on the differences and similarities in the findings for marmosets and macaques.

 Our Conclusion section is high level and does refer to the common marmoset data. However, in the revised paper we now state explicitly in the Conclusion (and Methods) that there were too few common marmoset data to generate a welfare assessment protocol for this species:

*There were too few marmoset data to generate a welfare assessment protocol for this species, and we recommend further data are collected.*

 Also in response to the reviewer, we have added marmosets to the second bullet under Research Highlights – Scientific benefits:

*Ranks welfare indicators for macaques and marmosets and narrows the field for further investigation of those considered most important by experts.*

 Regarding the comment on Table 2 (GEN-MAC protocol) indications of how frequently the observations of behavioural states and events would be carried out are given in the table (e.g. *on inspection or recorded in daily logs)*: these are suggestions only, to allow the GEN-MAC protocol to be adapted to local circumstances. They are based on the views of the Delphi participants, which are summarised in Table 4.

 We thank the reviewer again for his constructive feedback.
